# Urban sustainability and resilience: What the literature tells us about “lock-ins”?

**DOI:** 10.1007/s13280-022-01817-w

**Published:** 2022-12-15

**Authors:** Attila Buzási, Anna Csizovszky

**Affiliations:** Department of Environmental Economics and Sustainability, Budapest University of Technology and Economics, Műegyetem rkp. 3., H-1111 Budapest, Hungary

**Keywords:** Lock-in, Review, Urban sustainability, Urban resilience

## Abstract

Inherited system features and challenges that can hinder urban planning initiatives must be taken into consideration before a path towards a sustainable future can be established. By putting the lock-in effect under scrutiny, it is possible to gain valuable insight to emphasize positive lock-ins and to prevent maladaptation and unsustainable solutions. This paper aims to review the current trends of urban studies regarding sustainability, resilience, and the lock-in effect, focusing on both hot topics and mutual integration by following the Preferred Reporting Items for Systematic Reviews and Meta-Analyses for Scoping Reviews (PRISMA-ScR protocol) and analyzing the top-cited articles on these topics from 2015 to 2021 in the Web of Science database. Based on the revised literature, the potential lock-ins of climate-friendly and sustainable urban development are not adequately discussed. Moreover, while urban sustainability and resilience are often treated as overlapping areas, there is a lack of publications that carefully examine their interlinked long-term perspectives for any hindering effects.

**Supplementary Information:**

The online version contains supplementary material available at 10.1007/s13280-022-01817-w.

## Introduction

Cities have always been at the forefront of reinforcing change towards a sustainable future. This is even more the case today, with ever-changing climatic factors at play, making cities more vulnerable to these external conditions (IPCC 2021). Unfortunately, these climatic factors are only foreseen to become more unpredictable in the future. While urban studies have focused on urban sustainability since the early 2000s (Sharifi 2021), the focus of this research has shifted towards climate resilience in recent years (Klopfer et al. 2021; Guo et al. 2022). Due to dynamically changing external factors, we have seen an increasing demand for quantitative and qualitative analyses of urban resilience to facilitate designing less vulnerable and more resilient urban areas (Woodruff et al. 2021; Datola et al. 2022; Yin et al. 2022). As well as the obvious challenge, these changes in the technological and social–ecological systems also represent an opportunity to prepare for sustainable and resilient urban transformations (Egerer et al. 2021; Amirzadeh et al. 2022).

Urban resilience, mitigation concepts, and related applied analyses have become the cornerstones of current urban policies (Reckien et al. 2019; Pietrapertosa et al. 2021). To achieve the proposed mitigation and adaptation objectives, a change is required in the urban planning status quo, which is heavily burdened by the existence of path dependencies (Hurlimann et al. 2021b; Schindler and Dionisio 2021; Hanger-Kopp et al. 2022). Our cities are naturally path-dependent due to their infrastructures and various historical circumstances (van den Bergh 2020), but they also have a pivotal role in the global transition to sustainability (Wolfram 2016). To meet mitigation and adaptation requirements, it is crucial to address and prevent potential negative lock-ins (Newman 2020).

Additionally, the literature also mentions positive lock-ins (Ürge-Vorsatz et al. 2018). The term “lock-in” has been recognized by scholars for decades, but its implementation remains fairly scattered across other disciplines. A few examples of the lock-in phenomenon are notably present in behavioral economics (Barnes et al. 2004), such as cognitive lock-in in online retail competition (Johnson et al. 2003) or future lock-in to “should” and “want” choices (Rogers and Bazerman 2008). Furthermore, in the field of technology it was observed that the diffusion of a given technology can be limited—if not halted completely—by switching costs (Farrell and Klemperer 2007). In the case of eco-innovations (Cecere et al. 2014) or electric vehicles (Cowan and Hultén 1996) technologies that were difficult to change, firms, institutions, and society all had a role to play in preventing widespread diffusion. This role of the lock-in effect can also be easily grasped through the highly complex and interconnected urban infrastructure, and its impact on climate-related goals can be noteworthy (Gomez Echeverri 2018; Ramyar et al. 2021; Gao et al. 2022). Certain infrastructure elements lead to a significant load in the global carbon budget and eventuate path-dependent and locked-in cities by committing cities on a certain path of greenhouse gas (GHG) emissions (Unruh 2000; Erickson and Tempest 2015). It is important to mention that this is also the case for infrastructure elements that are yet to be built.

We apply the following definition for “lock-in” in our paper: lock-in is a path-dependent process that can result in future transformations being prohibited due to a set of favorable initial conditions and benefits. Path dependency refers to situations when the past has a significant impact on current choices and behavior (Drechsler and Wätzold 2020). Fundamentally, path dependence describes the idea that “history matters,” while the lock-in effect emphasizes the specific outcomes and illustrates how or why systems are difficult to modify (Cairns 2014).

As sharp GHG reduction targets and resilience-related efforts coincide with broader sustainability goals for urban areas, these aspects need to be integrated into current urban planning processes to avoid lock-ins and foster transformative change (A. Hurlimann et al. 2021a; Mehryar et al. 2022). The above processes are based on local futures and path dependencies, which must be integrated into everyday planning approaches in a precise manner (Cheung and Oßenbrügge 2020). It should be emphasized that analyzing mitigation and adaptation objectives and interventions with regard to their long-term impacts is not a new phenomenon in the literature (Boyd et al. 2022). In particular, numerous co-benefits of this approach can be identified, including a clearer view of the associated trade-offs and locked-in paths (Sharifi et al. 2021).

One specific objective of this study was to review the academic literature and examine the current research trends in the areas of sustainable development, resilience, and lock-in-related urban studies. Second, we aim to explore the interconnectedness of the three paradigms. The methodology applied is a widely accepted and used review process called the Preferred Reporting Items for Systematic Reviews and Meta-Analyses for Scoping Reviews (PRISMA-ScR) protocol (Tricco et al. 2018), which followed a rigorous scoping review process and laid the groundwork for further qualitative analysis of the selected papers. The remainder of this paper will firstly explain the applied methodology in great detail, followed by the results of the detailed paper analysis based on the PRISMA-ScR protocol (see Supplementary Information). Then, discussion remarks about the targeted study analysis and the conclusions can be found. Since the structure of this paper is defined by the PRISMA-ScR protocol, it requires a slightly different structure than that which research papers generally use. As a result, a section on the limitations of the study, the summary of evidence, and the conclusions can be found in the “Discussion and conclusions” section.

## Methodology

We have previously established a growing necessity for the simultaneous consideration of lock-ins, resilience, and sustainability. On that account, this paper aims to analyze the most cited documents on these topics from 2015 to 2021 and identify any potential literature gaps related to the interconnectedness of the paradigms, while also recommending other aspects to consider. There are two main approaches to this: one is to study the literature that uses and interprets the paradigms together. The downside of this approach is that it provides little information about the separate trends in the topics. The second approach is to examine sustainability, resilience, and lock-in urban studies separately, looking for overlaps and differences. We have opted for the latter approach. This research focuses on the areas of urban sustainability, resilience, lock-in, and the integration of these three fields in the urban context. In line with the purpose of our research to provide a broad and exploratory view, a scoping review was performed and reported by the PRISMA-ScR, see more: http://www.prisma-statement.org.

**Fig. 1:**
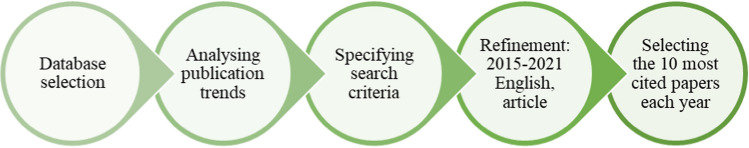
Sample selection process

The first step in this process (Fig. 1) was to select the appropriate database. In general, Google Scholar (GS), Scopus, and Web of Science (WoS) can be easily distinguished based on their key characteristics: GS tends to cover a more comprehensive range of sources, but their lower citation rate (owing to the significant proportion of non-journal and non-English results) indicates a lower scientific impact of the additional reporting (Martín-Martín et al. 2018). WoS and Scopus provide a broad range of peer-reviewed academic literature. However, unlike Scopus, WoS categories include urban studies and tend to be more accurate in classifying journals into research areas (Wang and Waltman 2016). To reveal and analyze the gaps in literature aiming to integrate urban sustainability, resilience, and lock-in, the WoS database was selected. Only studies previously categorized as “urban studies” were included.

**Fig. 2:**
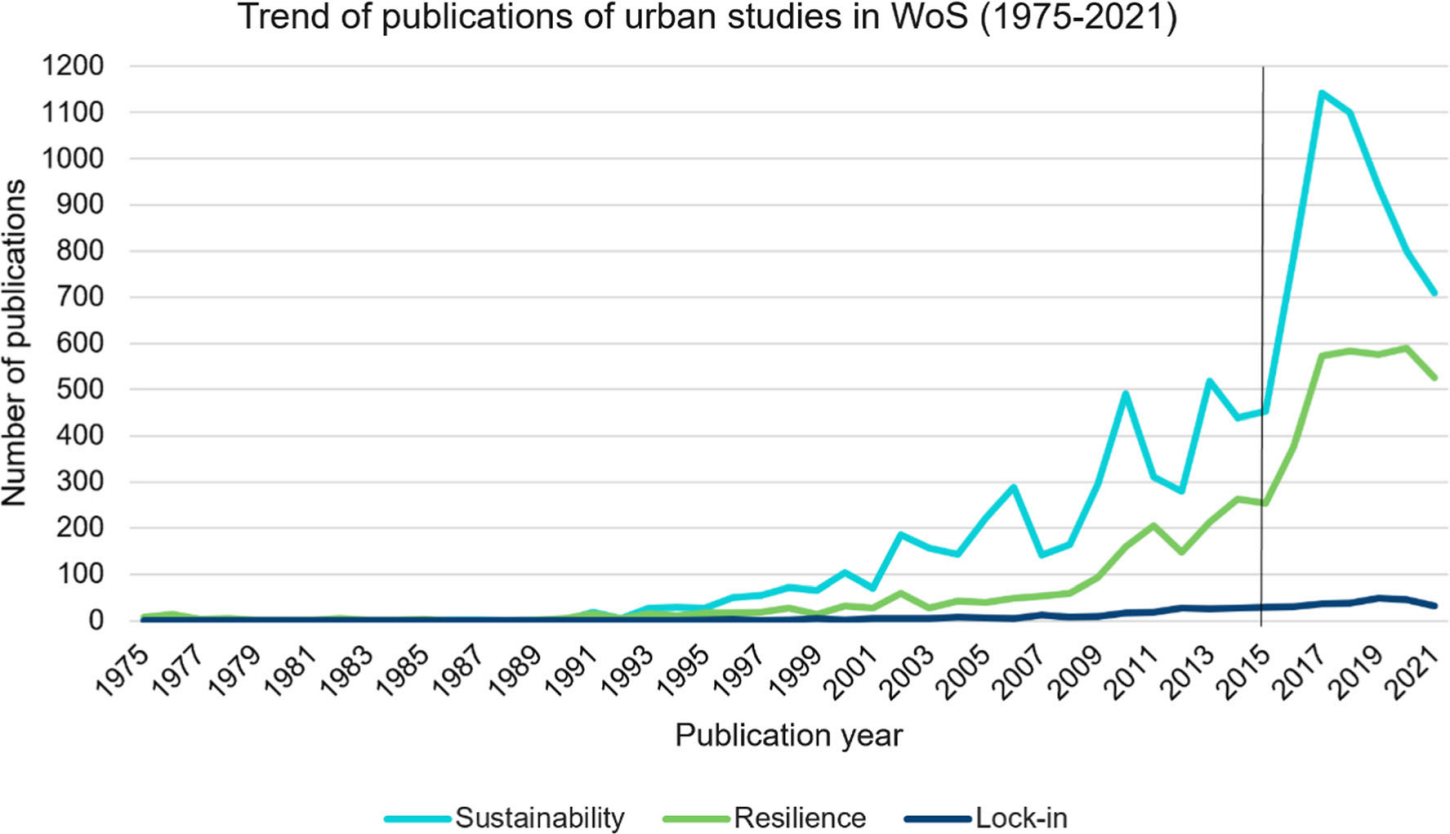
Publication trends of urban studies in WoS (1975–2021)

In the next step, we designed the exact search criteria for exclusion or inclusion. The category filter ensured a broad but topic-related scope of the academic literature, which enabled us to search not only by title but also by topic (title, keywords, and abstract). Compared with sustainability and resilience, we faced more difficulties choosing the proper parameters in the case of lock-in. The parameters have therefore been revised multiple times before reaching the final search criteria. The final search terms were “sustainab*” for sustainability, “resilien* or adapt*” for resilience, and “lock-in” or “path dependen*” or “lock in” or “locking in” or “embedded*” and “climate change” for lock-in, which resulted in 10 090, 5129, and 448 papers, respectively. After analyzing the publication trends, we identified a rapid growth in the number of sustainability and resilience-related documents after 2015, as shown in Fig. 2. Taking this into consideration, the analyzed period for each topic was limited to 2015–2021. Only peer-reviewed articles written in English were included, while books, editorial materials, and proceeding papers were excluded. Finally, the articles were filtered by citation due to the pivotal role of the most cited articles in influencing scientific trends (Teplitskiy et al. 2022) and their more interdisciplinary and diverse characteristics (Small 2018; Chen et al. 2021). Since the articles from 2015 to 2016 are cited much more frequently than those from 2021, a purely citation-based narrowing would have resulted in the exclusion of the more recent articles, preventing us from exploring the current trends. For a more comprehensive picture and to monitor possible changes over time, the ten most cited papers each year were selected for further examination, resulting in 70 articles per topic. In the case of citation equality, the authors chose the most relevant article for the search performed. An exception is the year 2021 in the topic of lock-in, where due to the limited number of papers (32) and the number of citations, the articles were prioritized according to relevance—the most cited article had only four citations at the time of the last search. These articles were checked for relevance, and incorrect results (e.g., articles mentioning adaptation only in terms of applying a method) were eliminated and replaced by upcoming articles; the authors discussed any uncertainty. The last search was carried out on 13 January 2022 (see the Supplementary Information for the final list of the selected papers).

All selected 189 papers (three times 70 minus 21 duplicates) were carefully read and subjected to qualitative analysis in the second phase. The following questions were raised to gain insights into research trends and draw conclusions regarding the simultaneous consideration of urban sustainability, adaptation, and the lock-in effect.

1. What are the main topics of the articles?
2. What are the main research questions, applied methodologies, scientific results, and conclusions of the selected papers?
3. Which of the studied paradigms are present, to what extent were they studied in-depth, and what definition was applied in the papers?

First, the abstracts were scanned to identify the main focus of each article (for instance, green infrastructure and land use in resilience-related studies). After analyzing one article together, the two authors worked independently and discussed the results by year and topic. If any questions arose during the evaluation, the article was checked independently by the other author and then discussed. The distinguished topic and subtopic categories have been reviewed extensively during the in-depth analysis process to find the most appropriate ones. Finally, the subtopics and research trends were illustrated through term co-occurrence networks created with VosViewer. VOSviewer (van Eck and Waltman 2010, 2017; https://www.vosviewer.com/) is a freely available software tool that allows bibliometric data (e.g., journals, citations, and authors) to be clustered and evaluated. The program relies heavily on visual representations to present the networks and additionally provides text-mining features. In this study, we applied the text mining function of VOSviewer to the titles and abstracts of the papers in all three themes differently. The output figures (see “Results”) show the different clusters (each with different colors); the size of the nodes indicates the number of occurrences, while the proximity between two nodes shows how often they occur together. It is worth noting that we have not questioned the appropriate use of the terms. For example, although many scholars have expressed doubts about whether smart city initiatives support the transition towards sustainability, we did not exclude indicator-focused smart city articles that stated contributing to sustainability as their main objective.

## Results

**Fig. 3:**
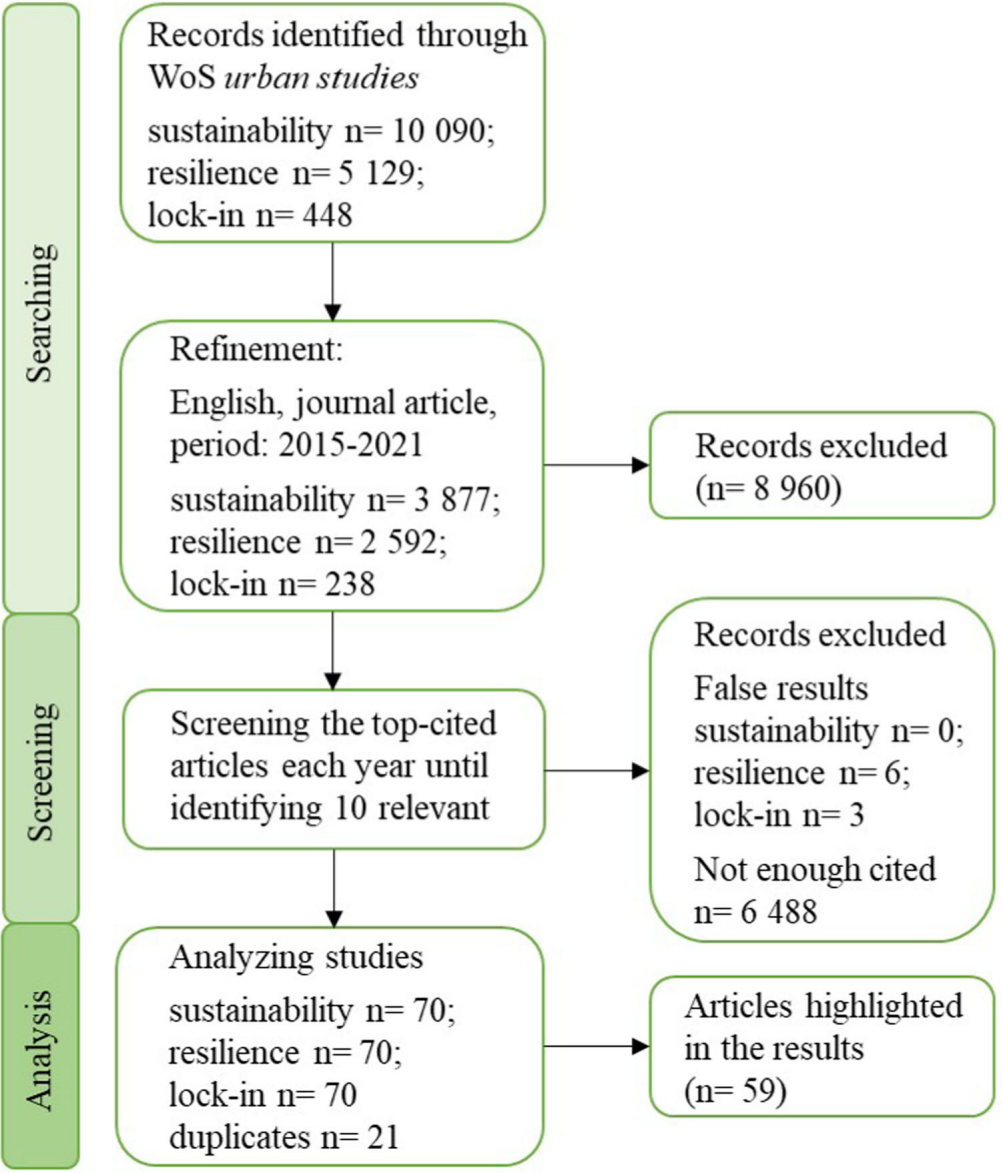
Selection of sources of evidence

To analyze the top-cited papers of current urban studies qualitatively, three research questions were defined (see “Methodology”), which can be answered after revealing the main features of the selected studies. In the following paragraphs, we have summarized the main results of the applied methodology based on the research questions. The number of sources reviewed, assessed for eligibility, and included in the scoping review processes can be found in Fig. 3. Once all subcategories were defined, they were illustrated through the term co-occurrence network in the title and abstract fields. The figures (created by VOSviewer) show the identified clusters, their “size,” and their overlap with other clusters. Moreover, the temporal changes regarding the analyzed topics are displayed. Finally, a detailed overview of each year and topics can be found in the Supplementary Information.

### What are the main topics of the articles?

**Fig. 4:**
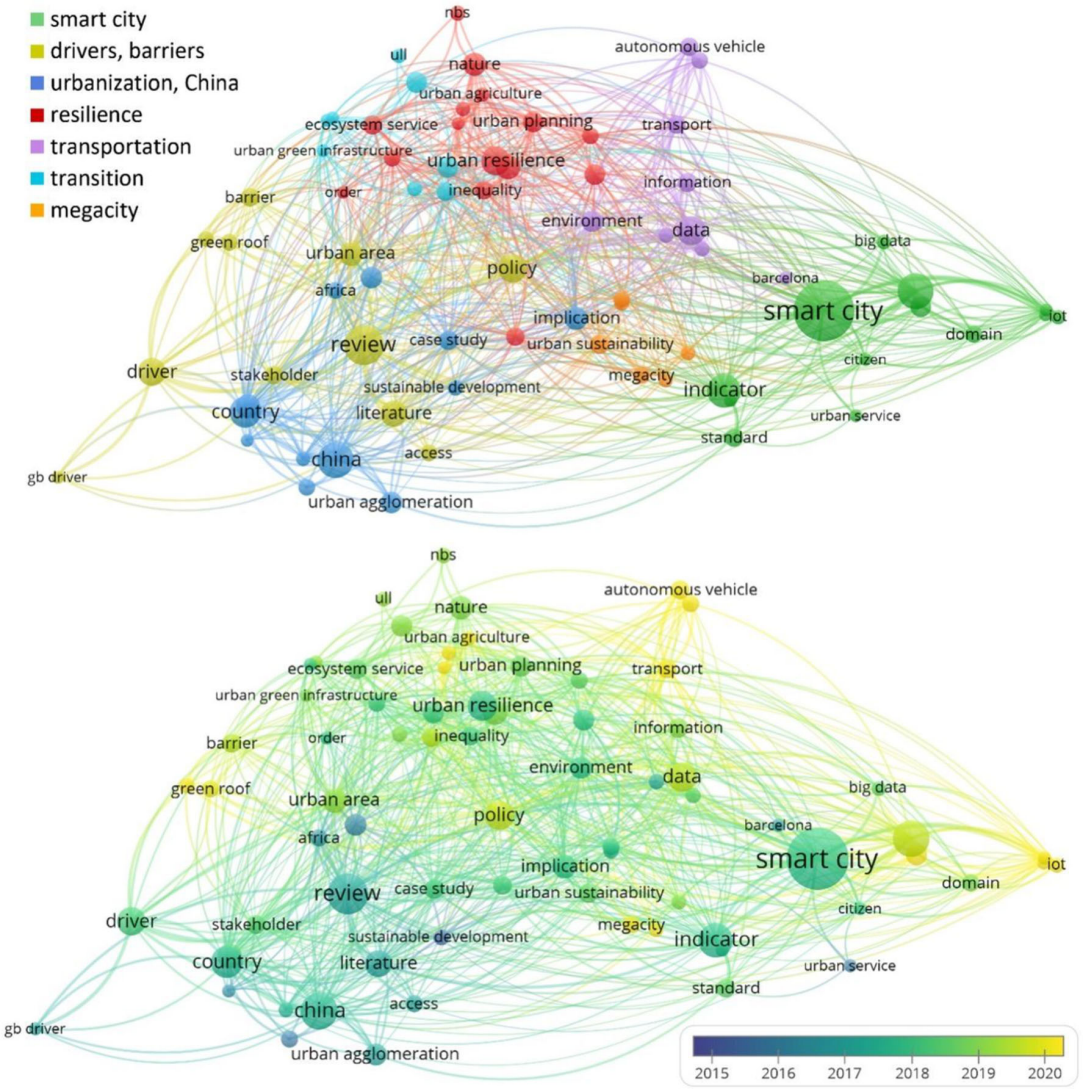
Term co-occurrence network of the most cited urban sustainability papers

The sustainability-focused papers encompass seven easily distinguishable topics identified through the analysis and illustrated by the term co-occurrence network: smart city, drivers and barriers, Chinese cities and urbanization, resilience, transportation, transition, and megacities (Fig. 4). The most highlighted aspects among the selected articles were smart city aspects and related applied indicator-based analysis, followed by country-level comparisons (mainly related to China). However, an interesting temporal dynamic can be identified in the questions asked by the top-cited papers: in the mid-2010s, the focus was on review articles and general sustainability aspects. Nonetheless, by the end of the decade, practice-oriented studies on smart and/or resilient cities have become the most influential. Although interdisciplinary topics, such as urban sustainability and urban resilience, are at the forefront of current urban studies, the trade-offs or co-benefits regarding the proposed interventions are rarely studied in sustainability-oriented articles. The selected papers of 2015 include only one study that discussed theoretical climate-related aspects. Chelleri et al. (2015) published one of the first ever manuscripts focusing on climate resilience, the lock-in effect, and the related sustainability challenges in urban areas. Kaika (2017) then considered potential path dependencies and lock-in effects in relation to smart and sustainable cities, which is almost entirely in contrast to what can be found in the literature from the previous year. Among the papers from 2018, Zhang and Li (2018) linked urban resilience and sustainability into a common theoretical framework that can address long-term path dependencies, lock-in opportunities, or other unintended adverse impacts of an improperly designed urban development action. In the late 2010s and early 2020s, Meerow and Newell (2019) provided a comprehensive theory of urban resilience with a hypothetical application to green infrastructure in the USA. Sharifi (2019) explored the role of urban forms in relation to the overall resilience of a given city, promoting the advantages of compact, polycentric, and landscape-connected cities. Finally, Olazabal and Gopegui (2021) strongly recommended integrating adaptation needs into the current urban development framework to ensure sustainable adaptation efforts.

**Fig. 5:**
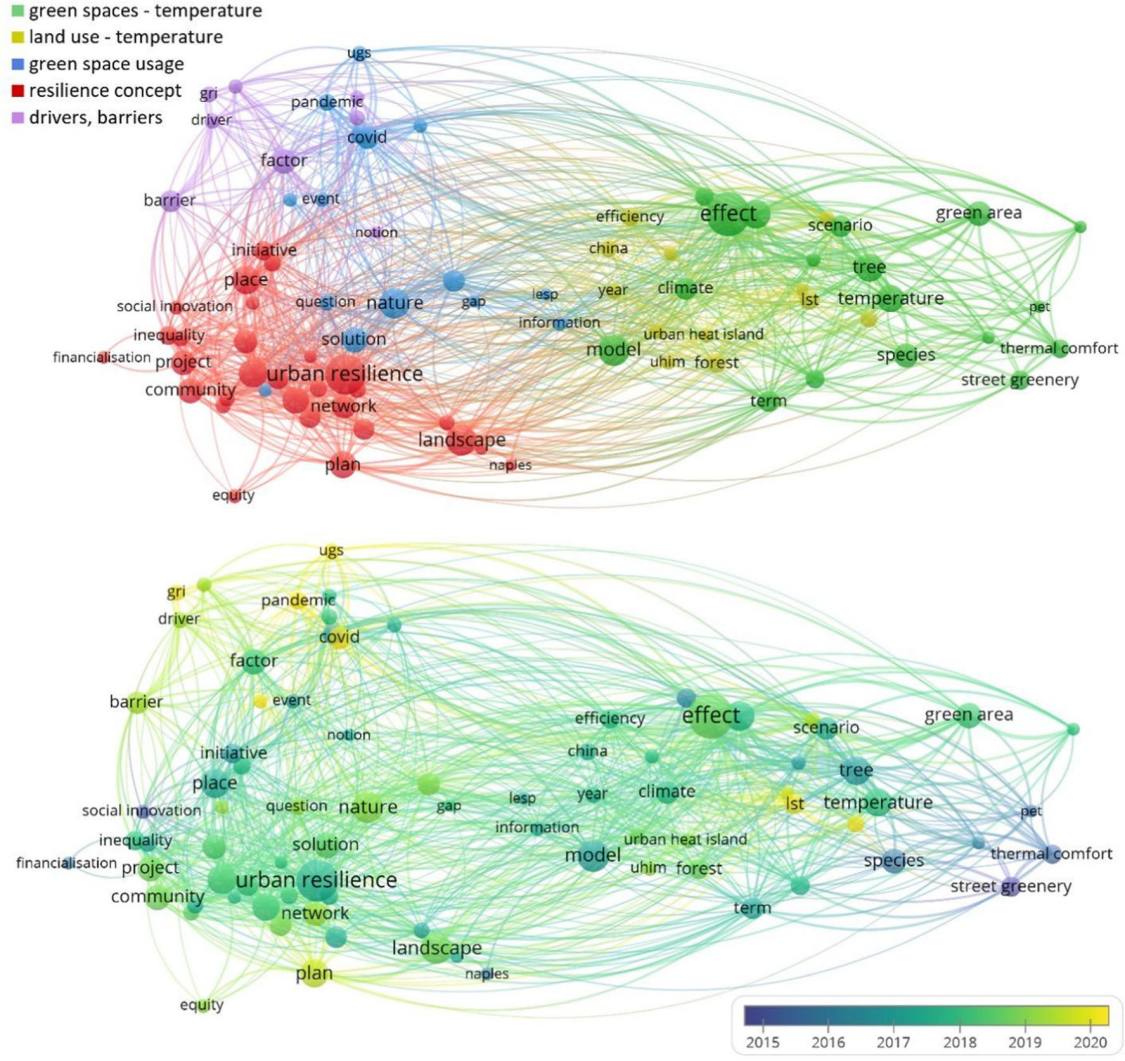
Term co-occurrence network of the most cited urban resilience papers

Those articles that primarily discuss urban resilience focused on infrastructure-related issues through green spaces and land-use patterns. Moreover, general concepts of resilience, as well as institutional drivers and barriers, can be identified as the most relevant issues (Fig. 5). Over time, the aforementioned infrastructure orientation shifted towards general concepts of resilience, then to the role of local communities and finally, to the importance of local specificities in the implementation phase. Anguelovski et al. (2016) showed that adaptation strategies could amplify socio–spatial inequalities, while Mehmood (2016) proposed an evolutionary resilience framework to address long-term challenges and emphasized the role of local communities and proactive planning. Similar to sustainability-based studies, the fact that potential trade-offs, co-benefits, path dependencies, or lock-ins cannot be distinguished among the hot topics in urban resilience studies indicates a gap for further future analysis. Among others, Meerow and Newell (2017) have proposed a framework for urban planning that facilitates the identification of high-priority areas for green infrastructure implementation. The framework also helps to address trade-offs and synergies. Meerow and Newell (2019) focused on the social and equity aspects of resilience planning. They presented a framework to identify trade-offs inherited from political and scalar difficulties. Similarly, Bush and Doyon (2019) developed a method to help urban planners address (temporal, scale, functional, social equity, and species) trade-offs, but they focused on nature-based solutions. However, at the end of the analyzed period, the emergence of the COVID-19 pandemic had an impact on urban resilience studies. Several papers addressed resilience in the face of the epidemic and stated that a radical change in urban planning is required. For example, Langemeyer et al. (2021) emphasized urban agriculture’s potential to contribute to resilience and sustainability as a nature-based multifunctional solution. Urban agriculture has lost its significance and they are calling for a revival. Moreno et al. (2021) introduced the concept of the 15-minute city and highlighted the importance of proximity and accessibility in urban planning. These overlapping issues might also indicate the rise of the importance of lock-in studies, since these interdisciplinary studies require a complex and long-term-oriented analysis, to consider path dependencies and long-term effects.

**Fig. 6:**
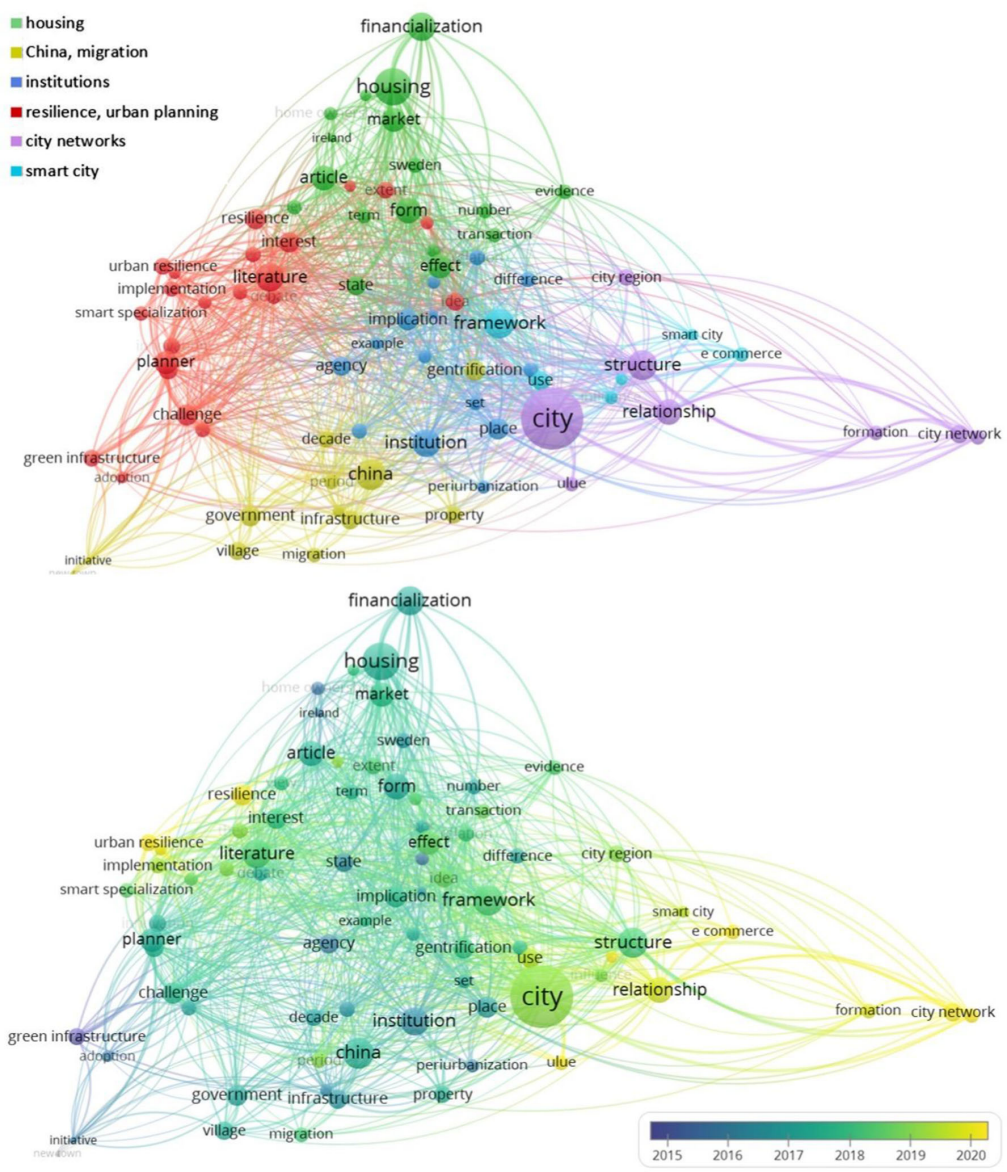
Term co-occurrence network of the most cited urban lock-in papers

Figure 6 shows the significant heterogeneity of identified topics and hot spots regarding the most cited lock-in-related papers. According to the analysis, six central elements can be distinguished: housing, migration and China, institutions, resilience, city networks, and smart cities. The housing studies are undoubtedly the most common, followed by migration-oriented and regional-focused papers. These aspects are overrepresented in lock-in papers and are less related to climate change issues. The first climate-related study in our database addressing potential lock-ins was written by Bouzarovski et al. (2016), who focused on energy poverty as a potentially path-dependent sector.

Furthermore, Kaika (2017) argues that finding path dependencies should be more emphasized in urban planning instead of focusing on different labels such as safe, sustainable, resilient, and inclusive. Only one selected article from 2018 focused on climate-related issues regarding lock-in analysis: Radhakrishnan et al. (2018) outlined the role of flexibility in adaptation planning processes in water-sensitive cities. In view of the change in these issues over time, no clearly recognizable trends can be identified. However, the emergence of resilience as a focus in the most-cited papers of 2020 (Goh 2020; Shamsuddin 2020; Wardekker et al. 2020; Ramos 2021) could be a sign of a shift in lock-in analyses toward more climate-related issues. Nevertheless, it can be stated that the analyzed articles on path dependency or lock-in focused on social aspects through housing studies and migration patterns. Thus, little attention was paid directly to urban sustainability or climate resilience aspects.

### What are the main research questions, applied methodologies, scientific results, and conclusions of the selected papers?

The selected sustainability-centered papers applied a wide range of methodologies and attempted to answer practical and planning-oriented questions, leading to practical results. In general, emerging theories such as smart cities and related technologies (e.g., Albino et al. 2015; Angelidou 2015; Belanche et al. 2016; Ahvenniemi et al. 2017; Yigitcanlar et al. 2018), and issues of urban sprawl (Ewing and Hamidi 2015) were studied, and comparative assessments on different scales were carried out numerically, providing quantitative results. In these cases, the results refer to a specific practice-oriented research question. These articles have almost nothing to do with path dependencies, climate-related issues, or potential locked-in features. In contrast, the papers that followed a theoretical approach presented and discussed contradictions in several aspects. They emphasized the role of considering the path-dependent and potentially locked-in urban features coupled with the identified co-benefits (Chelleri et al. 2015). These studies addressed various concepts of resilience, from climate-related vulnerability (Spaans and Waterhout 2017; Meerow and Newell 2019) to dependency on urban agriculture (Morgan 2015; Langemeyer et al. 2021) and COVID-19. Nevertheless, a scientific gap regarding the applied or quantitative analysis of the lock-in effect can be considered.

Among resilience-related urban studies, green infrastructure studies constitute another subcategory, and their early development was observed in the analyzed period. The main research questions were related to the thermal effects of different types of green spaces, the benefits of green infrastructure, urban planning implications, and residents’ usage patterns (du Toit et al. 2018; Pauleit et al. 2019; Ugolini et al. 2020). In general, each paper confirmed the positive contribution of green infrastructure to resilience, especially in the face of the pandemic. Regarding urban planning and resilience theory, the introduction of Sustainable Development Goal (SDG) 11 led to a steep increase in the number of critical papers related to business-as-usual methodologies, equity issues related to resilience, and the focus on sustainability indicators (Anguelovski et al. 2016; Caprotti et al. 2017; Klopp and Petretta 2017; Ziervogel et al. 2017). Consequently, some of the subsequent articles addressed the social justice of resilience hand in hand with sustainability. Resilience planning has also recently (particularly in 2019, before the Covid-19-related studies emerged) become more practice-oriented. These studies concentrate on drivers and barriers, thus indirectly alluding to the lock-in effect (Bush and Doyon 2019; Piggott-McKellar et al. 2019). Among the top-cited resilience papers, specific trends can be distinguished regarding not only topics but methodology as well. Half of the studies addressing green infrastructure implied quantitative methods to measure the heat mitigation effect of different green spaces (Klemm et al. 2015; Razzaghmanesh et al. 2016; Sodoudi et al. 2018; Yu et al. 2018; Nastran et al. 2019; Hou and Estoque 2020) or species (de Abreu-Harbich et al. 2015; Speak et al. 2020). These articles were popular throughout the studied period. Other papers focus on urban planning, social aspects, barriers, and drivers related to green infrastructure through frameworks or case studies (Matthews et al. 2015; McClintock et al. 2016; Derkzen et al. 2017; Meerow and Newell 2017). In 2020–2021 green infrastructure reviews appeared as well (Chatzimentor et al. 2020; Yu et al. 2020; Zhang and He 2021). The concept of resilience, urban planning, and—in most cases—green infrastructure appeared side by side in most qualitative studies, especially in the second half of the analysis period. Finally, it should be noted that only a few studies have applied a quantitative methodology to support urban planning decisions. Those papers aimed to enhance resilience through improved green infrastructure site selection (Meerow and Newell 2017; Li et al. 2020). This indicates a lack of theme–methodology coupling concerning the quantitative analysis of resilient urban planning decisions.

Finally, the lock-in-centered papers followed a strong practice-oriented approach to uncover path dependencies, mainly related to housing issues, regional clusters, and regional development. The focus was rarely on sustainability or climate-related aspects. Only a few papers—12 in total compared with the entire pool of selected studies—addressed these issues from the selected studies (Malekpour et al. 2015; Matthews et al. 2015; Bouzarovski et al. 2016; Gouldson et al. 2016; Bouzarovski and Tirado Herrero 2017; Kaika 2017; Radhakrishnan et al. 2018; Davidson et al. 2019; Goh 2020; Wardekker et al. 2020; Alaedini and Yeganeh 2021; Schindler and Dionisio 2021). However, the research questions were very practical given that the identified urban issues required scientifically proven solutions to serious social and economic challenges. Therefore, the results were quite local- or region-specific. Furthermore, these papers did not focus on overlapping issues or quantitative analysis of climate-related lock-in effects. In addition, the associated sustainability challenges were only mentioned to a limited extent. The analysis results were very focused and specific to the issues addressed. Regarding the lock-in effect, housing studies included both quantitative and qualitative methods, depending on whether they focused on infrastructure (earlier) or institutional (later) issues. None of the analyzed papers applied quantitative methods related to resilience and urban planning (due to their institutional focus). Regarding the expanded sustainability focus in 2016–2017, we found—somewhat unexpectedly—that mainly quantitative methods were implied. However, these studies addressed low-carbon transition (Gouldson et al. 2016) or energy poverty (Bouzarovski et al. 2016; Bouzarovski and Tirado Herrero 2017), not comprehensive sustainability issues.

### Which of the studied paradigms are present, to what extent were they studied in-depth, and what definition was applied in the papers?

The majority of selected urban sustainability studies up to 2017 are related to applied aspects, such as the smart technology implementation, land-use patterns, Chinese urbanization, etc., with no or minimal consideration of resilience or lock-in effects. As an interdisciplinary concept, sustainability has been incorporated into these articles by applying a narrower theoretical approach that focuses on various applied aspects as part of the broader discipline. However, as of 2017, the selected papers pointed to several conflicting issues about the long-term effects of originally sustainability-oriented urban development interventions. This trend continued from 2018, when resilience and path-dependency became a significant part of the urban studies discourse. With the spread of these highly overlapping but fundamentally different concepts (urban sustainability, resilience, and lock-in), their simultaneous consideration became essential. Thus, articles related to urban sustainability and resilience defined sustainability as the ultimate goal of urban development actions. However, the above aspects need to be considered and included in studies that attempt to capture long-term sustainability issues. Overall, the urban sustainability discourse has shifted significantly in the second half of the 2010s, looking at resilience and lock-ins in much more detail to improve and ensure broader sustainability. Nevertheless, it can be argued that most of the top-cited papers should pay more attention to defining and analyzing potential negative lock-ins to avoid maladaptation or unsustainable solutions, and even more to positive lock-ins that can lay the foundations for long-term sustainability.

More than a third of the resilience articles had little exposure to resilience, a common issue among the green infrastructure papers that analyzed land cover—temperature correlation and only mentioned adaptation in the broader context/potential application. Interestingly, resilience urban studies almost simultaneously referred to resilience with an emphasis on flexibility, adaptation, and transition in the face of short- and long-term changes. This is clearly in contrast to the frequent critics of resilience that the term is used with ambiguity. Meerow et al. (2016) provided the most comprehensive definition, meaning “the ability of an urban system—and all its constituent socio-ecological and socio-technical networks across temporal and spatial scales—to maintain or rapidly return to desired functions in the face of a disturbance, to adapt to change, and to quickly transform systems that limit current or future adaptive capacity.” Economic resilience is an exception, this emerging term means economic recovery (in the face of the pandemic) (Francke and Korevaar 2021; McCartney et al. 2021). Since the emergence of the new SDGs and the urban agenda, resilience studies (with few exceptions) also address sustainability, at least slightly as the more significant aim behind resilience or indirectly through equity issues. Interestingly, while urban planning-related adaptation papers do not tend to directly acknowledge lock-ins and path dependence, most consider them in an indirect manner. These studies predominantly focused on institutional lock-ins and sometimes included infrastructural/technological path dependencies. Institutional lock-ins were addressed through previous decision-making practices that—owing to their embeddedness in current planning—(negatively) affect the ability to respond to new challenges. Infrastructural path dependencies were considered in local specific characteristics, while technological ones referred to car dependence. However, resilience studies hardly addressed behavioral lock-ins, i.e., how residents’ persistent habits affect the feasibility of the initiative.

In lock-in-centered studies, there was a strong emphasis on institutional and infrastructural path dependencies, while behavioral studies, such as cultural fixes or habits, were under-emphasized. Institutional path dependencies were addressed from both positive and negative perspectives. Embedding flexibility, as a key resilience feature, in water-sensitive urban planning (Radhakrishnan et al. 2018) is a good example of (possible) positive lock-ins, as it can determine beneficial adaptation in the long run. Negative lock-ins were considered through business-as-usual practices, which proved insufficient in the case of, for example, suburban shrinkage (Ohashi and Phelps 2020) or rapid urbanization (Alaedini and Yeganeh 2021). Although most path dependency studies indirectly addressed sustainability through housing affordability, regional development, and urban sprawl, only a few deeply emphasized the paradigm. Sustainability-related path dependency articles explored low-carbon transition/energy poverty. In terms of resilience, lock-ins have played a central role in urban planning, primarily through institutional considerations.

## Discussion and conclusion

The present paper aims to analyze the top-cited papers in urban studies from 2015 to 2021 in the topics of lock-in, sustainability, and resilience through a scoping review guided by the standards of the PRISMA-ScR. The applied exploratory approach allowed us to examine current research trends along with the interconnectedness of the above three areas, as well as to identify potentially underrepresented areas. The selected articles showed great diversity in terms of their main questions, applied methodology, and results. The main issues were smart cities and urbanization in sustainability; green infrastructure, land-use, and urban planning in resilience; and housing, migration, and regional clusters in lock-in studies. As we have observed, any overlap between the three paradigms was only examined in the field of urban planning, and even these studies lack the aspect of analyzing the three paradigms simultaneously. Typically, the lock-in phenomenon in sustainability and resilience-oriented papers can be captured indirectly through inherited institutional processes, local specifics, or trade-offs. As such, most articles that explore the various aspects of urban planning, mention the lock-in phenomenon without going into greater detail regarding its specifics. However, few papers have focused directly on path dependencies, suggesting that the term needs better acknowledgment. In addition, behavioral aspects (e.g., how residents’ habits affect the outcomes of the initiatives) are unaccounted for. The integration of resilience and sustainability is in a much more advanced phase, due to the launch of SDG 11 (Make cities and human settlements inclusive, safe, resilient, and sustainable) and the New Urban Agenda. This trend is even more obvious in the years following 2017. In general, sustainability is the “larger, long-term goal” behind resilient adaptations. Besides deeper integration of the lock-in phenomenon, we have identified another shortcoming in urban planning studies: the lack of quantitative approaches to support (ex-ante) or evaluate (ex-post) decision-making. While acknowledging the difficulties related to such frameworks and data collection, we make a case for the potential long-term benefits.

Analyzing the topics and main research questions, the presence of land-use issues in the areas of sustainability, resilience, and lock-in is striking. However, these studies focus on specific practical issues and do not adequately consider integration with the other domains. Sustainability-focused papers explored urbanization in terms of land-use characteristics, resilience-focused studies investigated correlations between temperature and land-use, while lock-in-centric articles analyzed land-use issues through the lens of inherited infrastructure and urban structures. An integration of the above areas would benefit land-use studies by providing more comprehensive results and a wider range of implications for decision-makers.

Focusing only on the most cited articles categorized as urban studies can lead to underestimating the importance of lock-in, a more common phenomenon in engineering and natural sciences. However, our study concentrated on the urban context and the integration of lock-in, sustainability-, and resilience-related urban research. It examined the cutting-edge, most cited literature as the most influential and important research segment. To ensure that our attention remains within the domain of urban studies, certain key terms that are mainly used in engineering fields (such as gridlock or inertia) were excluded from our search in the case of lock-ins. As mentioned above, the applied methodology has certain limitations, such as the possibility that relevant publications may be overlooked throughout the evidence-gathering process due to missing citations or research categories. Furthermore, the focus on broad exploratory research questions does not include an in-depth analysis of the overlaps, i.e., the results of studies that have specifically looked at sustainability, resilience, and lock-in together. Consequently, this paper differs markedly from the previously cited well-known articles in the research area. The main conclusions are based on a holistic overview of the selected topics rather than focusing on case studies. This differentiated view enabled us to compare the mutual embedding of urban sustainability, resilience, and lock-in from a macro perspective and to focus on horizontal aspects.

Apart from the theoretically oriented thoughts on the results of our study, through the mutually reinforcing mechanisms, carbon lock-ins play a crucial role in urban management processes by delaying further transitions (Seto et al. 2016). In parallel with the identification of path-dependent trajectories related to urban transformation, the lock-in effect has received considerable attention from researchers (Ürge-Vorsatz et al. 2018), paving the way for further research in the area for years and even decades to come. Timely recognition, analysis, and prevention of negative lock-ins actively contribute to societal transformations, making them crucial steps to achieve several SDGs. Infrastructural lock-ins can hinder sustainability transitions related to SDGs 6 (Clean water and sanitation), 7 (Affordable and clean energy), 9 (Industry, innovation and infrastructure), 11 (Sustainable cities and communities), and 13 (Climate action).

In addition, behavioral lock-ins can have negative long-term effects on SDGs 3 (Good health and well-being), 4 (Quality education), and 12 (Responsible consumption and production). Finally, institutional lock-ins can hinder the achievement of SDGs 1 (No poverty), 2 (Zero hunger), 5 (Gender equality), 10 (Reduced inequalities), 14 (Life on land), 15 (Life below water), and 16 (Peace, justice, and strong institutions). As urban areas play a central role in sustainability transitions and the achievement of many SDGs, policymakers need to consider lock-ins in strategy development and evaluation processes.

In summary, the integration of lock-in analyses into urban sustainability and resilience studies is still in its infancy. However, all these highly interdisciplinary concepts unquestionably require the incorporation and in-depth analysis of long-term impacts and inherited system characteristics alongside barriers and trade-offs to avoid unsustainable solutions or maladaptation. A possible explanation for the lack of mutual embedding is the different connotation of the words. Sustainability and resilience inherently have a positive meaning through the desired outcomes of climate-friendly and sustainable urban development processes. For example, the SDGs include specific goals for humanity that help improve the well-being of both developed and developing countries and their societies. Still, the term “lock-in” is mainly used with a negative connotation to refer to the phenomenon of being trapped in undesirable situations with little chance of escape. The simultaneous consideration of these aspects is, therefore, only present to a limited extent even in the most frequently cited urban studies. To avoid long-term harmful consequences, mainstreaming lock-in assessments should be a future research direction of urban studies, regardless of the often negative connotations of the word “lock-in.”

## Supplementary Information

Below is the link to the electronic supplementary material.

Supplementary file1 (PDF 460 kb)
