# Supplementary material for: Urban sustainability and resilience: What the literature tells us about “lock-ins”?
Source: Ambio. 2022 Dec 15;52(3):616–30. doi: 10.1007/s13280-022-01817-w (PMC9753863; doi:10.1007/s13280-022-01817-w)
Supplement: Supplementary file 1 — Supplementary file1 (PDF 460 kb) [file 13280_2022_1817_MOESM1_ESM.pdf]

Ambio

Supplementary Information

This supplementary information has not been peer reviewed.

Title: **Urban sustainability and resilience: What the literature tells us about "lock-ins"?**

Table S1. Research inputs used during the scoping review

SUSTAINABILITY

Searching parameters: sustainab* (Topic) and 2021 or 2020 or 2019 or 2018 or 2017 or 2016 or 2015 (Publication Years) and Urban Studies (Web of Science Categories) [13/01/2022]

Authors	Title	Type	Year	Source
Albino, V; Berardi, U; Dangelico, RM	Smart Cities: Definitions, Dimensions, Performance, and Initiatives	Article	2015	Journal of Urban Technology
Haaland, C; van den Bosch, CK	Challenges and strategies for urban green-space planning in cities undergoing densification: A review	Review	2015	Urban Forestry & Urban Greening
Angelidou, M	Smart cities: A conjuncture of four forces	Article	2015	Cities
de Abreu-Harbicha, LV; Labakia, LC; Matzarakis, A	Effect of tree planting design and tree species on human thermal comfort in the tropics	Article	2015	Landscape and Urban Planning
Chelleri, L; Waters, JJ; Olazabal, M; Minucci, G	Resilience trade-offs: addressing multiple scales and temporal aspects of urban resilience	Article	2015	Environment and Urbanization
Forsyth, A	What is a walkable place? The walkability debate in urban design	Article	2015	Urban Design International
Cobbinah, PB; Erdiaw-Kwasie, MO; Amoateng, P	Africa's urbanisation: Implications for sustainable development	Article	2015	Cities
Morgan, K	Nourishing the city: The rise of the urban food question in the Global North	Article	2015	Urban Studies
Ewing, R; Hamidi, S	Compactness versus Sprawl: A Review of Recent Evidence from the United States	Article	2015	Journal of Planning Literature
Homsy, GC; Warner, ME	Cities and Sustainability: Polycentric Action and Multilevel Governance	Review	2015	Urban Affairs Review
Rigolon, A	A complex landscape of inequity in access to urban parks: A literature review	Review	2016	Landscape and Urban Planning
Darko, A; Chan, APC	Critical analysis of green building research trend in construction journals	Review	2016	Habitat International
Tan, YT; Xu, H; Zhang, XL	Sustainable urbanization in China: A comprehensive literature review	Article	2016	Cities
March, H; Ribera-Fumaz, R	Smart contradictions: The politics of making Barcelona a Self-sufficient city	Article	2016	European Urban and Regional Studies
Wei, YG; Huang, C; Li, J; Xie, LL	An evaluation model for urban carrying capacity: A case study of China's mega-cities	Article	2016	Habitat International
Zhang, XQ	The trends, promises and challenges of urbanisation in the world	Article	2016	Habitat International
Belanche, D; Casalo, LV; Orus, C	City attachment and use of urban services: Benefits for smart cities	Article	2016	Cities

Barnett, C; Parnell, S	Ideas, implementation and indicators: epistemologies of the post-2015 urban agenda	Article	2016	Environment and Urbanization
Mao, C; Xie, FY; Hou, L; Wu, P; Wang, J; Wang, XY	Cost analysis for sustainable off-site construction based on a multiple-case study in China	Article	2016	Habitat International
Huang, L; Yan, LJ; Wu, JG	Assessing urban sustainability of Chinese megacities: 35 years after the economic reform and open-door policy	Article	2016	Landscape and Urban Planning
Ahvenniemi, H; Huovila, A; Pinto-Seppa, I; Airaksinen, M	What are the differences between sustainable and smart cities?	Article	2017	Cities
Fang, CL; Yu, DL	Urban agglomeration: An evolving concept of an emerging phenomenon	Article	2017	Landscape and Urban Planning
Haase, D; Kabisch, S; Haase, A; Andersson, E; Banzhaf, E; Baro, F; Brenck, M; Fischer, LK; Frantzeskaki, N; Kabisch, N; Krellenberg, K; Kremer, P; Kronenberg, J; Larondelle, N; Mathey, J; Pauleit, S; Ring, I; Rink, D; Schwarz, N; Wolff, M	Greening cities - To be socially inclusive? About the alleged paradox of society and ecology in cities	Article	2017	Habitat International
Stevens, MR	Does Compact Development Make People Drive Less?	Article	2017	Journal of The American Planning Association
Kaika, M	Don't call me resilient again!': the New Urban Agenda as immunology ... or ... what happens when communities refuse to be vaccinated with smart cities' and indicators	Article	2017	Environment and Urbanization
Darko, A; Zhang, CZ; Chan, APC	Drivers for green building: A review of empirical studies	Review	2017	Habitat International
Klopp, JM; Petretta, DL	The urban sustainable development goal: Indicators, complexity and the politics of measuring cities	Article	2017	Cities
Aflaki, A; Mirnezhad, M; Ghaffarianhoseini, A; Ghaffarianhoseini, A; Omrany, H;	Urban heat island mitigation strategies: A state-of-the-art review on Kuala Lumpur, Singapore and Hong Kong	Review	2017	Cities

Wang, ZH; Akbari, H				
Anthopoulos, L	Smart utopia VS smart reality: Learning by experience from 10 smart city cases	Article	2017	Cities
Spaans, M; Waterhout, B	Building up resilience in cities worldwide - Rotterdam as participant in the 100 Resilient Cities Programme	Article	2017	Cities
Yigitcanlar, T; Kamruzzaman, M; Buys, L; Ioppolo, G; Sabatini-Marques, J; da Costa, EM; Yun, JJ	Understanding 'smart cities': Intertwining development drivers with desired outcomes in a multidimensional framework	Article	2018	Cities
Guan, XL; Wei, HK; Lu, SS; Dai, Q; Su, HJ	Assessment on the urbanization strategy in China: Achievements, challenges and reflections	Article	2018	Habitat International
Ruhlandt, RWS	The governance of smart cities: A systematic literature review	Review	2018	Cities
Lim, C; Kim, KJ; Maglio, PP	Smart cities with big data: Reference models, challenges, and considerations	Article	2018	Cities
Allam, Z; Newman, P	Redefining the Smart City: Culture, Metabolism and Governance	Article	2018	Smart Cities
He, BJ; Zhao, DX; Zhu, J; Darko, A; Gou, ZH	Promoting and implementing urban sustainability in China: An integration of sustainable initiatives at different urban scales	Article	2018	Habitat International
Anguelovski, I; Connolly, JJT; Masip, L; Pearsall, H	Assessing green gentrification in historically disenfranchised neighborhoods: a longitudinal and spatial analysis of Barcelona	Article	2018	Urban Geography
Zhang, XL; Li, H	Urban resilience and urban sustainability: What we know and what do not know?	Article	2018	Cities
Li, L; Bergen, JM	Green infrastructure for sustainable urban water management: Practices of five forerunner cities	Article	2018	Cities
du Toit, MJ; Cilliers, SS; Dallimer, M; Goddard, M; Guenat, S; Cornelius, SF	Urban green infrastructure and ecosystem services in sub-Saharan Africa	Article	2018	Landscape and Urban Planning
Meerow, S; Newell, JP	Urban resilience for whom, what, when, where, and why?	Article	2019	Urban Geography
Allam, Z; Dhunny, ZA	On big data, artificial intelligence and smart cities	Article	2019	Cities
Huovila, A; Bosch, P; Airaksinen, M	Comparative analysis of standardized indicators for Smart sustainable cities: What indicators and standards to use and when?	Article	2019	Cities
Albert, C; Schroter, B;	Addressing societal challenges through nature-based solutions: How can	Article	2019	Landscape and Urban Planning

Haase, D; Brillinger, M; Henze, J; Herrmann, S; Gottwald, S; Guerrero, P; Nicolas, C; Matzdorf, B	landscape planning and governance research contribute?			
von Wirth, T; Fuenfschilling, L; Frantzeskaki, N; Coenen, L	Impacts of urban living labs on sustainability transitions: mechanisms and strategies for systemic change through experimentation	Article	2019	European Planning Studies
Joss, S; Sengers, F; Schraven, D; Caprotti, F; Dayot, Y	The Smart City as Global Discourse: Storylines and Critical Junctures across 27 Cities	Article	2019	Journal Of Urban Technology
Pauleit, S; Ambrose-Oji, B; Andersson, E; Anton, B; Buijs, A; Haase, D; Elands, B; Hansen, R; Kowarik, I; Kronenberg, J; Mattijssen, T; Olafsson, AS; Rall, E; van der Jagt, APN; van den Bosch, CK	Advancing urban green infrastructure in Europe: Outcomes and reflections from the GREEN SURGE project	Review	2019	Urban Forestry & Urban Greening
Bush, J; Doyon, A	Building urban resilience with nature-based solutions: How can urban planning contribute?	Article	2019	Cities
Yigitcanlar, T; Foth, M; Kamruzzaman, M	Towards Post-Anthropocentric Cities: Reconceptualizing Smart Cities to Evade Urban Ecocide	Article	2019	Journal of Urban Technology
Sharifi, A	Resilient urban forms: A macro-scale analysis	Article	2019	Cities
Corbera, E; Anguelovski, I; Honey-Roses, J; Ruiz-Mallen, I	Academia in the Time of COVID-19: Towards an Ethics of Care	Article	2020	Planning Theory & Practice
Galli, A; Iha, K; Pires, SM; Mancini, MS; Alves, A; Zokai, G; Lin, D; Murthy, A; Wackernagel, M	Assessing the Ecological Footprint and biocapacity of Portuguese cities: Critical results for environmental awareness and local management	Article	2020	Cities
Rice, JL; Cohen, DA; Long, J; Jurjevich, JR	Contradictions of the Climate-Friendly City: New Perspectives on Eco-Gentrification and Housing Justice	Article	2020	International Journal of Urban and Regional Research

Xia, C; Yeh, AGO; Zhang, AQ	Analyzing spatial relationships between urban land use intensity and urban vitality at street block level: A case study of five Chinese megacities	Article	2020	Landscape And Urban Planning
Yigitcanlar, T; Kankanamge, N; Vella, K	How Are Smart City Concepts and Technologies Perceived and Utilized? A Systematic Geo-Twitter Analysis of Smart Cities in Australia	Article	2020	Journal of Urban Technology
Kronenberg, J; Haase, A; Laszkiewicz, E; Antal, A; Baravikova, A; Biernacka, M; Dushkova, D; Filcak, R; Haase, D; Ignatieva, M; Khmara, Y; Nita, MR; Onose, DA	Environmental justice in the context of urban green space availability, accessibility, and attractiveness in postsocialist cities	Article	2020	Cities
Heikinheimo, V; Tenkanen, H; Bergroth, C; Jarv, O; Hiippala, T; Toivonen, T	Understanding the use of urban green spaces from user-generated geographic information	Article	2020	Landscape and Urban Planning
Li, LY; Uyttenhove, P; Vaneetvelde, V	Planning green infrastructure to mitigate urban surface water flooding risk - A methodology to identify priority areas applied in the city of Ghent	Article	2020	Landscape and Urban Planning
Meerow, S	The politics of multifunctional green infrastructure planning in New York City	Article	2020	Cities
Shafique, M; Xue, XL; Luo, XW	An overview of carbon sequestration of green roofs in urban areas	Review	2020	Urban Forestry & Urban Greening
Moreno, C; Allam, Z; Chabaud, D; Gall, C; Pratlong, F	Introducing the 15-Minute City: Sustainability, Resilience and Place Identity in Future Post-Pandemic Cities	Article	2021	Smart Cities
Langemeyer, J; Madrid-Lopez, C; Beltran, AM; Mendez, GV	Urban agriculture? A necessary pathway towards urban resilience and global sustainability?	Article	2021	Landscape and Urban Planning
Hussain, T; Abbas, J; Wei, Z; Ahmad, S; Bi, XH; Zhu, GL	Impact of Urban Village Disamenity on Neighboring Residential Properties: Empirical Evidence from Nanjing through Hedonic Pricing Model Appraisal	Article	2021	Journal of Urban Planning and Development
Acheampong, RA; Cugurullo, F; Gueriau, M; Dusparic, I	Can autonomous vehicles enable sustainable mobility in future cities? Insights and policy challenges from user preferences over different urban transport options	Article	2021	Cities

Zhang, GC; He, BJ	Towards green roof implementation: Drivers, motivations, barriers and recommendations	Review	2021	Urban Forestry & Urban Greening
Syed, AS; Sierra-Sosa, D; Kumar, A; Elmaghraby, A	IoT in Smart Cities: A Survey of Technologies, Practices and Challenges	Article	2021	Smart Cities
Olazabal, M; De Gopegui, MR	Adaptation planning in large cities is unlikely to be effective	Article	2021	Landscape and Urban Planning
Zhou, Q; Zhu, MK; Qiao, YR; Zhang, XL; Chen, J	Achieving resilience through smart cities? Evidence from China	Article	2021	Habitat International
Song, XQ; Feng, Q; Xia, FZ; Li, XY; Scheffran, J	Impacts of changing urban land-use structure on sustainable city growth in China: A population-density dynamics perspective	Article	2021	Habitat International
Su, SL; Zhang, JY; He, SJ; Zhang, H; Hu, LR; Kang, MJ	Unraveling the impact of TOD on housing rental prices and implications on spatial planning: A comparative analysis of five Chinese megacities	Article	2021	Habitat International

RESILIENCE

Searching parameters: resilien* or adapt* (Topic) and 2021 or 2020 or 2019 or 2018 or 2017 or 2016 or 2015 (Publication Years) and Urban Studies (Web of Science Categories) [13/01/2022]

Authors	Title	Type	Year	Source
Norton, BA; Coutts, AM; Livesley, SJ; Harris, RJ; Hunter, AM; Williams, NSG	Planning for cooler cities: A framework to prioritise green infrastructure to mitigate high temperatures in urban landscapes	Article	2015	Landscape and Urban Planning
Matthews, T; Lo, AY; Byrne, JA	Reconceptualizing green infrastructure for climate change adaptation: Barriers to adoption and drivers for uptake by spatial planners	Article	2015	Landscape and Urban Planning
de Abreu-Harbicha, LV; Labakia, LC; Matzarakis, A	Effect of tree planting design and tree species on human thermal comfort in the tropics	Article	2015	Landscape and Urban Planning
Chelleri, L; Waters, JJ; Olazabal, M; Minucci, G	Resilience trade-offs: addressing multiple scales and temporal aspects of urban resilience	Article	2015	Environment and Urbanization
Klemm, W; Heusinkveld, BG; Lenzholzer, S; van Hove, B	Street greenery and its physical and psychological impact on thermal comfort	Article	2015	Landscape and Urban Planning
Barthel, S; Parker, J; Ernstson, H	Food and Green Space in Cities: A Resilience Lens on Gardens and Urban Environmental Movements	Article	2015	Urban Studies

Chen, WY	The role of urban green infrastructure in offsetting carbon emissions in 35 major Chinese cities: A nationwide estimate	Article	2015	Cities
Lujala, P; Lein, H; Rod, JK	Climate change, natural hazards, and risk perception: the role of proximity and personal experience	Article	2015	Local Environment
Baker, S; Mehmood, A	Social innovation and the governance of sustainable places	Article	2015	Local Environment
Berke, P; Newman, G; Lee, J; Combs, T; Kolosna, C; Salvesen, D	Evaluation of Networks of Plans and Vulnerability to Hazards and Climate Change	Article	2015	Journal of The American Planning Association
Meerow, S; Newell, JP; Stults, M	Defining urban resilience: A review	Review	2016	Landscape and Urban Planning
Anguelovski, I; Shi, LD; Chu, E; Gallagher, D; Goh, K; Lamb, Z; Reeve, K; Teicher, H	Equity Impacts of Urban Land Use Planning for Climate Adaptation: Critical Perspectives from the Global North and South	Article	2016	Journal of Planning Education and Research
Fields, D; Uffer, S	The financialisation of rental housing: A comparative analysis of New York City and Berlin	Article	2016	Urban Studies
Zolch, T; Maderspacher, J; Wamsler, C; Pauleit, S	Using green infrastructure for urban climate-proofing: An evaluation of heat mitigation measures at the micro-scale	Article	2016	Urban Forestry & Urban Greening
Razzaghmanesh, M; Beecham, S; Salemi, T	The role of green roofs in mitigating Urban Heat Island effects in the metropolitan area of Adelaide, South Australia	Article	2016	Urban Forestry & Urban Greening
Leitner, H; Sheppard, E	Provincializing Critical Urban Theory: Extending the Ecosystem of Possibilities	Article	2016	International Journal of Urban and Regional Research
De Rosa, S; Salvati, L	Beyond a 'side street story'? Naples from spontaneous centrality to entropic polycentricism, towards a 'crisis city'	Article	2016	Cities
Sjoman, H; Morgenroth, J; Sjoman, JD; Saebo, A; Kowarik, I	Diversification of the urban forest-Can we afford to exclude exotic tree species?	Article	2016	Urban Forestry & Urban Greening
Mehmood, A	Of resilient places: planning for urban resilience	Article	2016	European Planning Studies
McClintock, N; Mahmoudi, D; Simpson, M; Santos, JP	Socio-spatial differentiation in the Sustainable City: A mixed-methods assessment of residential gardens in metropolitan Portland, Oregon, USA	Article	2016	Landscape and Urban Planning
Liu, XP; Liang, X; Li, X; Xu, XC;	A future land use simulation model (FLUS) for simulating multiple land	Article	2017	Landscape and Urban Planning

Ou, JP; Chen, YM; Li, SY; Wang, SJ; Pei, FS	use scenarios by coupling human and natural effects			
Meerow, S; Newell, JP	Spatial planning for multifunctional green infrastructure: Growing resilience in Detroit	Article	2017	Landscape and Urban Planning
Kaika, M	Don't call me resilient again!': the New Urban Agenda as immunology ... or ... what happens when communities refuse to be vaccinated with smart cities' and indicators	Article	2017	Environment and Urbanization
Klopp, JM; Petretta, DL	The urban sustainable development goal: Indicators, complexity and the politics of measuring cities	Article	2017	Cities
Ziervogel, G; Pelling, M; Cartwright, A; Chu, E; Deshpande, T; Harris, L; Hyams, K; Kaunda, J; Klaus, B; Michael, K; Pasquini, L; Pharoah, R; Rodina, L; Scott, D; Zweig, P	Inserting rights and justice into urban resilience: a focus on everyday risk	Article	2017	Environment and Urbanization
Anthopoulos, L	Smart utopia VS smart reality: Learning by experience from 10 smart city cases	Article	2017	Cities
Derkzen, ML; van Teeffelen, AJA; Verburg, PH	Green infrastructure for urban climate adaptation: How do residents' views on climate impacts and green infrastructure shape adaptation preferences?	Article	2017	Landscape and Urban Planning
Spaans, M; Waterhout, B	Building up resilience in cities worldwide - Rotterdam as participant in the 100 Resilient Cities Programme	Article	2017	Cities
Caprotti, F; Cowley, R; Datta, A; Broto, VC; Gao, E; Georgeson, L; Herrick, C; Odendaal, N; Joss, S	The New Urban Agenda: key opportunities and challenges for policy and practice	Article	2017	Urban Research & Practice
Zhang, LQ; Peng, J; Liu, YX; Wu, JS	Coupling ecosystem services supply and human ecological demand to identify landscape ecological security pattern: A case study in Beijing-Tianjin-Hebei region, China	Article	2017	Urban Ecosystems
Allam, Z; Newman, P	Redefining the Smart City: Culture, Metabolism and Governance	Article	2018	Smart Cities

Zhang, XL; Li, H	Urban resilience and urban sustainability: What we know and what do not know?	Article	2018	Cities
Peng, J; Pan, YJ; Liu, YX; Zhao, HJ; Wang, YL	Linking ecological degradation risk to identify ecological security patterns in a rapidly urbanizing landscape	Article	2018	Habitat International
du Toit, MJ; Cilliers, SS; Dallimer, M; Goddard, M; Guenat, S; Cornelius, SF	Urban green infrastructure and ecosystem services in sub-Saharan Africa	Article	2018	Landscape and Urban Planning
Tu, SS; Long, HL; Zhang, YN; Ge, DZ; Qu, Y	Rural restructuring at village level under rapid urbanization in metropolitan suburbs of China and its implications for innovations in land use policy	Article	2018	Habitat International
Yu, ZW; Guo, XY; Zeng, YX; Koga, M; Vejre, H	Variations in land surface temperature and cooling efficiency of green space in rapid urbanization: The case of Fuzhou city, China	Article	2018	Urban Forestry & Urban Greening
Langemeyer, J; Camps-Calvet, M; Calvet-Mir, L; Barthel, S; Gomez-Baggethun, E	Stewardship of urban ecosystem services: understanding the value(s) of urban gardens in Barcelona	Article	2018	Landscape and Urban Planning
Sodoudi, S; Zhang, HW; Chi, XL; Muller, F; Li, HD	The influence of spatial configuration of green areas on microclimate and thermal comfort	Article	2018	Urban Forestry & Urban Greening
Leitner, H; Sheppard, E; Webber, S; Colven, E	Globalizing urban resilience	Article	2018	Urban Geography
Fischer, AP	Forest landscapes as social-ecological systems and implications for management	Article	2018	Landscape and Urban Planning
Meerow, S; Newell, JP	Urban resilience for whom, what, when, where, and why?	Article	2019	Urban Geography
von Wirth, T; Fuenfschilling, L; Frantzeskaki, N; Coenen, L	Impacts of urban living labs on sustainability transitions: mechanisms and strategies for systemic change through experimentation	Article	2019	European Planning Studies
Pauleit, S; Ambrose-Oji, B; Andersson, E; Anton, B; Buijs, A; Haase, D; Elands, B; Hansen, R; Kowarik, I; Kronenberg, J; Mattijssen, T;	Advancing urban green infrastructure in Europe: Outcomes and reflections from the GREEN SURGE project	Review	2019	Urban Forestry & Urban Greening

Olafsson, AS; Rall, E; van der Jagt, APN; van den Bosch, CK				
Bush, J; Doyon, A	Building urban resilience with nature-based solutions: How can urban planning contribute?	Article	2019	Cities
Nastran, M; Kobal, M; Eler, K	Urban heat islands in relation to green land use in European cities	Article	2019	Urban Forestry & Urban Greening
Sharifi, A	Resilient urban forms: A macro-scale analysis	Article	2019	Cities
Long, J; Rice, JL	From sustainable urbanism to climate urbanism	Article	2019	Urban Studies
Meerow, S; Pajouhesh, P; Miller, TR	Social equity in urban resilience planning	Article	2019	Local Environment
Abu Hatab, A; Cavinato, MER; Lindemer, A; Lagerkvist, CJ	Urban sprawl, food security and agricultural systems in developing countries: A systematic review of the literature	Review	2019	Cities
Piggott-McKellar, AE; McNamara, KE; Nunn, PD; Watson, JEM	What are the barriers to successful community-based climate change adaptation? A review of grey literature	Article	2019	Local Environment
Ugolini, F; Massetti, L; Calaza-Martinez, P; Carinanos, P; Dobbs, C; Ostoic, SK; Marin, AM; Pearlmutter, D; Saaroni, H; Sauliene, I; Simoneti, M; Verlic, A; Vuletic, D; Sanesi, G	Effects of the COVID-19 pandemic on the use and perceptions of urban green space: An international exploratory study	Article	2020	Urban Forestry & Urban Greening
Yu, ZW; Yang, GY; Zuo, SD; Jorgensen, G; Koga, M; Vejre, H	Critical review on the cooling effect of urban blue-green space: A threshold-size perspective	Review	2020	Urban Forestry & Urban Greening
Boeing, G	A multi-scale analysis of 27,00 urban street networks: Every US city, town, urbanized area, and Zillow neighborhood	Article	2020	Environment and Planning B-Urban Analytics and City Science
Li, LY; Uyttenhove, P; Vaneetvelde, V	Planning green infrastructure to mitigate urban surface water flooding risk - A methodology to identify priority areas applied in the city of Ghent	Article	2020	Landscape and Urban Planning
Chatzimentor, A; Apostolopoulou, E; Mazaris, AD	A review of green infrastructure research in Europe: Challenges and opportunities	Review	2020	Landscape and Urban Planning

Speak, A; Montagnani, L; Wellstein, C; Zerbe, S	The influence of tree traits on urban ground surface shade cooling	Article	2020	Landscape and Urban Planning
Goh, K	Flows in formation: The global-urban networks of climate change adaptation	Article	2020	Urban Studies
Hou, H; Estoque, RC	Detecting Cooling Effect of Landscape from Composition and Configuration: An Urban Heat Island Study on Hangzhou	Article	2020	Urban Forestry & Urban Greening
Wardekker, A; Wilk, B; Brown, V; Uittenbroek, C; Mees, H; Driessen, P; Wassen, M; Molenaar, A; Walda, J; Runhaar, H	A diagnostic tool for supporting policymaking on urban resilience	Article	2020	Cities
Loughran, K	Urban parks and urban problems: An historical perspective on green space development as a cultural fix	Article	2020	Urban Studies
Moreno, C; Allam, Z; Chabaud, D; Gall, C; Pratlong, F	Introducing the 15-Minute City: Sustainability, Resilience and Place Identity in Future Post-Pandemic Cities	Article	2021	Smart Cities
Langemeyer, J; Madrid-Lopez, C; Beltran, AM; Mendez, GV	Urban agriculture? A necessary pathway towards urban resilience and global sustainability?	Article	2021	Landscape and Urban Planning
Zhang, GC; He, BJ	Towards green roof implementation: Drivers, motivations, barriers and recommendations	Review	2021	Urban Forestry & Urban Greening
McCartney, G; Pinto, J; Liu, M	City resilience and recovery from COVID-19: The case of Macao	Article	2021	Cities
Francke, M; Korevaar, M	Housing markets in a pandemic: Evidence from historical outbreaks	Article	2021	Journal of Urban Economics
Olazabal, M; De Gopegui, MR	Adaptation planning in large cities is unlikely to be effective	Article	2021	Landscape and Urban Planning
Masik, G; Sagan, I; Scott, JW	Smart City strategies and new urban development policies in the Polish context	Article	2021	Cities
Shi, YJ; Zhai, GF; Xu, LH; Zhou, ST; Lu, YW; Liu, HB; Huang, W	Assessment methods of urban system resilience: From the perspective of complex adaptive system theory	Article	2021	Cities
Cao, J; Zhou, WQ; Zheng, Z; Ren, T; Wang, WM	Within-city spatial and temporal heterogeneity of air temperature and its relationship with land surface temperature	Article	2021	Landscape and Urban Planning
Birchall, SJ; Bonnett, N	Climate change adaptation policy and practice: The role of agents, institutions and systems	Article	2021	Cities

LOCK-IN

Searching parameters: lock-in or ‘path dependen*’ or ‘lock in’ or ‘locking in’ or embedded* and ‘climate change’ (Topic) and 2021 or 2020 or 2019 or 2018 or 2017 or 2016 or 2015 (Publication Years) and Urban Studies (Web of Science Categories) [13/01/2022]

Authors	Title	Type	Year	Source
Matthews, T; Lo, AY; Byrne, JA	Reconceptualizing green infrastructure for climate change adaptation: Barriers to adoption and drivers for uptake by spatial planners	Article	2015	Landscape and Urban Planning
Kroll, H	Efforts to Implement Smart Specialization in Practice-Leading Unlike Horses to the Water	Article	2015	European Planning Studies
Stephens, M; Lux, M; Sunega, P	Post-Socialist Housing Systems in Europe: Housing Welfare Regimes by Default?	Article	2015	Housing Studies
Trippl, M; Grillitsch, M; Isaksen, A; Sinozic, T	Perspectives on Cluster Evolution: Critical Review and Future Research Issues	Review	2015	European Planning Studies
Malekpour, S; Brown, RR; de Haan, FJ	Strategic planning of urban infrastructure for environmental sustainability: Understanding the past to intervene for the future	Article	2015	Cities
Li, LY	State rescaling and national new area development in China: The case of Chongqing Liangjiang	Article	2015	Habitat International
Martin, H; Coenen, L	Institutional Context and Cluster Emergence: The Biogas Industry in Southern Sweden	Article	2015	European Planning Studies
Haughton, G; Allmendinger, P	Fluid Spatial Imaginaries: Evolving Estuarial City-regional Spaces	Article	2015	International Journal of Urban and Regional Research
Grillitsch, M	Institutional Layers, Connectedness and Change: Implications for Economic Evolution in Regions	Article	2015	European Planning Studies
Filion, P	Suburban Inertia: The Entrenchment of Dispersed Suburbanism	Article	2015	International Journal of Urban and Regional Research
Shin, HB	Economic transition and speculative urbanisation in China: Gentrification versus dispossession	Article; Proceedings Paper	2016	Urban Studies
Wolfram, M	Conceptualizing urban transformative capacity: A framework for research and policy	Article	2016	Cities

Authors	Title	Type	Year	Source
Gouldson, A; Colenbrander, S; Sudmant, A; Papargyropoulou, E; Kerr, N; McAnulla, F; Hall, S	Cities and climate change mitigation: Economic opportunities and governance challenges in Asia	Article	2016	Cities
Affolderbach, J; Schulz, C	Mobile transitions: Exploring synergies for urban sustainability research	Article	2016	Urban Studies
Bouzarovski, S; Herrero, ST; Petrova, S; Urge-Vorsatz, D	Unpacking the spaces and politics of energy poverty: path-dependencies, deprivation and fuel switching in post-communist Hungary	Article	2016	Local Environment
Haurin, D; Ma, C; Moulton, S; Schmeiser, M; Seligman, J; Shi, W	Spatial Variation in Reverse Mortgages Usage: House Price Dynamics and Consumer Selection	Article	2016	Journal of Real Estate Finance and Economics
Zukauskaite, E; Moodysson, J	Multiple paths of development: knowledge bases and institutional characteristics of the Swedish food sector	Article	2016	European Planning Studies
Norris, M	Varieties of Home Ownership: Ireland's Transition from a Socialised to a Marketised Policy Regime	Article	2016	Housing Studies
Sorensen, A	Periurbanization as the institutionalization of place: The case of Japan	Article	2016	Cities
Hytonen, J; Mantysalo, R; Peltonen, L; Kanninen, V; Niemi, P; Simanainen, M	Defensive routines in land use policy steering in Finnish urban regions	Article	2016	European Urban and Regional Studies
Xiao, Y; Wang, Z; Li, ZG; Tang, ZL	An assessment of urban park access in Shanghai - Implications for the social equity in urban China	Article	2017	Landscape and Urban Planning
Kaika, M	Don't call me resilient again!': the New Urban Agenda as immunology ... or ... what happens when communities refuse to be vaccinated with smart cities' and indicators	Article	2017	Environment and Urbanization
Aalbers, MB	The Variegated Financialization of Housing	Article	2017	International Journal of Urban and Regional Research
Bouzarovski, S; Herrero, S	The energy divide: Integrating energy transitions, regional inequalities and poverty trends in the European Union	Article	2017	European Urban and Regional Studies
Berger, T; Enflo, K	Locomotives of local growth: The short-and long-term impact of railroads in Sweden	Article	2017	Journal of Urban Economics

Authors	Title	Type	Year	Source
Guo, Y; Xiao, Y; Yuan, QF	The redevelopment of peri-urban villages in the context of path-dependent land institution change and its impact on Chinese inclusive urbanization: The case of Nanhai, China	Article	2017	Cities
Pereira, ALD	Financialization of Housing in Brazil: New Frontiers	Article	2017	International Journal of Urban and Regional Research
Puustinen, S; Mantysalo, R; Hytonen, J; Jarenko, K	The deliberative bureaucrat: deliberative democracy and institutional trust in the jurisdiction of the Finnish planner	Article	2017	Planning Theory & Practice
Webb, B; Webber, S	The implications of condominium neighbourhoods for long-term urban revitalisation	Article	2017	Cities
Amars, L; Fridahl, M; Hagemann, M; Roser, F; Linner, BO	The transformational potential of Nationally Appropriate Mitigation Actions in Tanzania: assessing the concept's cultural legitimacy among stakeholders in the solar energy sector	Article	2017	Local Environment
Grillitsch, M; Asheim, B	Place-based innovation policy for industrial diversification in regions	Article	2018	European Planning Studies
Sorensen, A	Institutions and Urban Space: Land, Infrastructure, and Governance in the Production of Urban Property	Article	2018	Planning Theory & Practice
Cao, Z; Zheng, XY; Liu, YS; Li, YR; Chen, YF	Exploring the changing patterns of China's migration and its determinants using census data of 2000 and 2010	Article	2018	Habitat International
Hein, C	Oil Spaces: The Global Petroleumscape in the Rotterdam/The Hague Area	Article	2018	Journal of Urban History
Radhakrishnan, M; Pathirana, A; Ashley, RM; Gersonius, B; Zevenbergen, C	Flexible adaptation planning for water sensitive cities	Article	2018	Cities
Sorvoll, J; Bengtsson, B	The Pyrrhic victory of civil society housing? Co-operative housing in Sweden and Norway	Article	2018	International Journal of Housing Policy
Gunko, M; Bogacheva, P; Medvedev, A; Kashnitsky, I	Path-Dependent Development of Mass Housing in Moscow, Russia	Article; Book Chapter	2018	Housing Estates in Europe: Poverty, Ethnic Segregation and Policy Challenges
Graca, M; Queiros, C; Farinha-Marques, P; Cunha, M	Street trees as cultural elements in the city: Understanding how perception affects ecosystem services management in Porto, Portugal	Article	2018	Urban Forestry & Urban Greening

Authors	Title	Type	Year	Source
Isaksen, A; Kyllingstad, N; Rypestol, JO; Schulze-Krogh, AC	Differentiated regional entrepreneurial discovery processes. A conceptual discussion and empirical illustration from three emergent clusters	Article	2018	European Planning Studies
Lazzeretti, L; Oliva, S	Rethinking city transformation: Florence from art city to creative fashion city	Article	2018	European Planning Studies
Hassink, R; Gong, HW	Six critical questions about smart specialization	Article	2019	European Planning Studies
Taubenbock, H; Gerten, C; Rusche, K; Siedentop, S; Wurm, M	Patterns of Eastern European urbanisation in the mirror of Western trends - Convergent, unique or hybrid?	Article	2019	Environment and Planning B-Urban Analytics and City Science
Blackwell, T; Kohl, S	Historicizing housing typologies: beyond welfare state regimes and varieties of residential capitalism	Article	2019	Housing Studies
Soaita, AM; Dewilde, C	A Critical-Realist View of Housing Quality within the Post-Communist EU States: Progressing towards a Middle-Range Explanation	Article	2019	Housing Theory & Society
Chen, HS; Wang, XP	Exploring the relationship between rural village characteristics and Chinese return migrants' participation in farming: Path dependence in rural employment	Article	2019	Cities
Davidson, K; Nguyen, TMP; Beilin, R; Briggs, J	The emerging addition of resilience as a component of sustainability in urban policy	Article	2019	Cities
Zhu, J; Jin, WW; He, CF	On evolutionary economic geography: a literature review using bibliometric analysis	Review	2019	European Planning Studies
Wang, T; Kazak, J; Han, Q; de Vries, B	A framework for path-dependent industrial land transition analysis using vector data	Article	2019	European Planning Studies
Murphy, L	Performing calculative practices: residual valuation, the residential development process and affordable housing	Article	2019	Housing Studies
Fritzsche, C; Vandrei, L	The German real estate transfer tax: Evidence for single-family home transactions	Article	2019	Regional Science and Urban Economics
Goh, K	Flows in formation: The global-urban networks of climate change adaptation	Article	2020	Urban Studies

Authors	Title	Type	Year	Source
Wardekker, A; Wilk, B; Brown, V; Uittenbroek, C; Mees, H; Driessen, P; Wassen, M; Molenaar, A; Walda, J; Runhaar, H	A diagnostic tool for supporting policymaking on urban resilience	Article	2020	Cities
Shamsuddin, S	Resilience resistance: The challenges and implications of urban resilience implementation	Article	2020	Cities
Ohashi, H; Phelps, NA	Diversity in decline: The changing suburban fortunes of Tokyo Metropolis	Article	2020	Cities
Jiao, LM; Xu, ZB; Xu, G; Zhao, R; Liu, JF; Wang, WL	Assessment of urban land use efficiency in China: A perspective of scaling law	Article	2020	Habitat International
Kubes, J; Kovacs, Z	The kaleidoscope of gentrification in post-socialist cities	Article	2020	Urban Studies
Janoschka, M; Mota, F	New municipalismin actionor urban neoliberalisationreloaded? An analysis of governance change, stability and path dependence in Madrid (2015-2019)	Article	2020	Urban Studies
Lee, A; Mackenzie, A; Smith, GJD; Box, P	Mapping Platform Urbanism: Charting the Nuance of the Platform Pivot	Article	2020	Urban Planning
Chong, ZH; Pan, S	Understanding the structure and determinants of city network through intra-firm service relationships: The case of Guangdong-Hong Kong-Macao Greater Bay Area	Article	2020	Cities
Kim, S	Inter-municipal relations in city-region governance	Article	2020	Cities
Yang, ZS; Wu, D; Wang, DW	Exploring spatial path dependence in industrial space with big data: A case study of Beijing	Article	2021	Cities
Schindler, M; Dionisio, R	A framework to assess impacts of path dependence on urban planning outcomes, induced through the use of decision-support tools	Article	2021	Cities
Morales, DAC; Dube, J	The evolution and trajectories of the geography of mergers and acquisitions: A city network analysis for Canada, 1994-2016	Article; Early Access	2021	Journal of Urban Affairs
Uyttebrouck, C; De Decker, P; Teller, J	Ontologies of live-work mix in Amsterdam, Brussels and Stockholm: an institutionalist approach drawing on path dependency	Article; Early Access	2021	European Planning Studies

Authors	Title	Type	Year	Source
Alaedini, P; Yeganeh, N	New-town programs and housing schemes: a case of mutual path dependence in Iran	Article; Early Access	2021	Journal of Housing and the Built Environment
Nahiduzzaman, KM; Holland, M; Sikder, SK; Shaw, P; Hewage, K; Sadiq, R	Urban Transformation Toward a Smart City: An E-Commerce-Induced Path-Dependent Analysis	Article	2021	Journal of Urban Planning and Development
Heaphy, L; Scott, M	Path dependence, 'lock-in' and rural housing outcomes: insights from Ireland	Article; Early Access	2021	European Planning Studies
Ramos, SJ	Resilience, Path Dependence, and the Port: The Case of Savannah	Article	2021	Journal of Urban History
Zhou, Y; Hu, CR	Industrial policy and differentiated regional diversifications: Evidence from Chinese cities	Article	2021	Cities
Aguda, D; Leishman, C	Neighbourhood Effects, Social Capital and Young Adults' Homeownership Outcomes in the United Kingdom	Article	2021	Housing Theory & Society

S2. Chronological overview of the revised literature - grouped by the selected topics

SUSTAINABILITY

2015

In the middle of the 2010s, the most cited papers focused on two easily distinguished topics - smart city and compact vs. sprawled areas - and some other horizontal issues. The first group can be formulated by considering the smart city-oriented. They dealt with smart technologies and the smart city itself as a potential solution for modern sustainable cities. The second large group of the selected and most cited literature is about various planning perspectives, indicating the considerable importance of practice-oriented urban studies in the 21st century. The selected papers of 2015 include only one study that dealt with horizontal climate-related aspects; Chelleri et al. (2015) published one of the very first manuscripts focusing on climate resilience, the lock-in effect, and the related sustainability challenges in urban areas. Their pioneer paper contributes to formulating the scientific discourse about such complex issues, which tries to solve highly interconnected aspects of urban sustainability.

2016

The most cited papers regarding urban sustainability issues indicate that there is a growing body of literature that recognizes the importance of horizontal aspects of highly local-specific sustainability challenges. They argued that small-scale pilot projects are unquestionably needed before increasing the scale of sustainable urban development interventions besides distinguishing those institutional barriers that can hamper the transformation processes; thus, a scale-related horizontal study has been involved in the most cited documents this year well after 2015. The second group of most cited papers from this year encompasses applied studies concerning green buildings and the construction sector; moreover, case studies in the field of assessment of urban sustainability by using macro indicators in the case of Chinese megacities and the analysis of urban carrying capacity. Finally, smart cities and smart interventions were in focus in several collected studies. Summarizing, it can be stated that the most cited urban sustainability papers from 2016 indicated a strong China-orientation, while case studies regarding the numerical analysis of broader urban sustainability or sector-oriented analyses are also among the top articles in this year; however, the resilience-sustainability framework can not be found at all.

2017

In 2017, an interesting trend can be defined among the top-cited articles on the topic since several studies emphasized the contradiction of previously discussed aspects. For example, Haase et al. (2017) argued the indisputably positive meaning of promoting green infrastructure and nature-based solutions throughout the city, while it can lead to less socially inclusive public spaces in the future. Similarly, Stevens (2017) discussed the possible adverse effects of compact cities, contributing to increased driving time instead of decreasing it due to the higher concentration of people, services, etc.. Kaika (2017) considered potential path dependencies and lock-in effects regarding smart and sustainable cities, which is almost totally contrary to

what can be found in the literature in the previous year. Besides the hereby introduced critical way, two selected articles dealt with the integration of smart and sustainable cities; finally, a considerable group of the studies chosen from this year includes highly practice-oriented papers.

2018

The 2018 trends regarding the mentioned and studied topics within urban sustainability is an exciting mix of the previously introduced aspects from 2015 and 2016. The papers can be grouped into four main bodies. The first one encompasses those studies which dealt with smart city issues and urban sustainability in a general way. The second group of studies includes those articles which focused on green areas from different perspectives; the third large group covers those studies which focused on Chinese urbanization aspects. Finally, Zhang and Li (2018) connected urban resilience and urban sustainability into a common theoretical framework that can tackle long-term path-dependencies, lock-in opportunities, or other non-intended adverse effects of a not carefully developed urban development action.

2019

2019 is the first year when the ten top-cited articles in urban sustainability studies include at least two papers with a solid urban resilience focus. Meerow and Newell (2019) provided a comprehensive urban resilience theory with a hypothetical case of green infrastructure in the U.S. to demonstrate the importance of trade-offs and their spatial and temporal features. Similarly, trade-offs were in the focus of the second resilience-based urban sustainability study written by Bush and Doyon (2019), who dealt with green infrastructure within that topic. Finally, Sharifi (2019) explored the role of urban forms regarding the overall resilience of a given city by arguing the advantages of compact, polycentric, and landscape-connected cities. The rest of the top-cited studies indicate a shift in urban sustainability studies since they all concentrated on the implementation phase; moreover, four articles of the ten analyzed studies were about different aspects of smart cities or utilization of different types of data.

2020

The most cited papers of 2020 represent considerable heterogeneity regarding the discussed topics, while new and highly complex aspects, such as climate change issues, can be found as well. Rice et al. (2020) argued the contradictions of climate-friendly cities and think critically about climate mitigation and adaptation issues within a broader sustainability narrative. Their article joined to that narrative in recent years about potential trade-offs, path-dependencies, and lock-ins of sustainable urban development interventions from a climate-friendly point of view. As it could be seen in the previous years, social aspects and environmental justice regarding urban green and blue spaces attracted considerable scientific interest. Contrary to the trends observed in last years, only one article dealt with smart city aspects. Finally, several implication-oriented papers have also been involved in the top articles, which assessed the benefits of green infrastructure projects and green.

2021

The 2021 collection of top-cited urban sustainability-oriented papers include those that can attract the most citations in the initial phase of their lives. Therefore, the following short introduction provides only a snapshot instead of a state-of-the-art literature review from 2021. Like 2016 and 2018, many papers focused on Chinese cities as case studies. Moreover, several studies carried on the trend of evaluating the implementation of smart city technologies as a significant dimension of sustainable cities. Finally, the largest group of top-cited articles focused on resilience aspects from very different perspectives: Moreno et al., (2021) proposed the 15-minute city which can be resilient enough to the external disturbances, such as the COVID pandemic; Langemeyer et al. (2021) identified urban agriculture as a cornerstone of broader resilience and sustainability through increasing the currently low-level food sovereignty of urban areas; Olazabal and Gopegui (2021) highly recommended the integration of adaptation needs in the current urban development framework to ensure sustainable adaptation efforts; finally, Zhou et al. (2021) opened a new discourse in the top-cited urban sustainability studies by introducing the term smart-resilient cities.

RESILIENCE

2015

In 2015 most papers dealt with green infrastructure; half of them quantified their cooling effects or carbon storage and sequestration, while others focused on the policy or conceptual aspects. Other studies take an emphasis on the concept and implementations of resilience considering the possibilities to broaden resilience from the narrow-minded climate adaptation and catastrophe management perspective to comprehend better global sustainability issues (Chelleri et al., 2015) or elaborate a resilience scoreboard to enhance the integration of local plans (Berke et al., 2015). Baker and Mehmood (2015) investigated social innovations and claimed that they could enhance locally specified sustainability and adaptive transition - it requires a certain level of well-being, needs for social change, and the integrated role of the public/private institutions, civil society, and citizens.

2016

Research on green infrastructure remained in focus in 2016; two out of four were quantitative studies concerning urban heat islands, while the remaining ones focused on the importance of considering non-native species in urban greening and explored the spatial and social differences in residential urban agriculture. Three articles dealt with the resilience concept: a comprehensive literature review (Meerow et al., 2016), which is highly the most cited urban resilience paper in the last six years. The other two approached the concept from the aspect of urban planning. On the one hand, Anguelovski et al. (2016) showed that adaptation strategies could intensify the socio-spatial inequalities, while on the other hand, Mehmood (2016) proposed an evolutionary resilience framework to address long-term challenges and relies on proactive planning and local communities. One of the remaining three studies is about the

consequences of private equity real estate investments on rental housing markets. Finally, the last two studies are concerned with urbanization from the approach of and practice regarding the effects of crises on urban development.

2017

The main focus shifted from green infrastructure to the conceptualization of resilience/ SDG 11 and urban planning in 2017 as a consequence of the introduction of the USDG (in 2015) and the New Urban Agenda (in 2016). Three highly cited papers (out of five categorized into 'concept') put the New Urban Agenda into their focus, forms critics concerning inequities, and path-dependence (Kaika, 2017), questions the indicator-based methodology and the urban focus or highlights challenges regarding indicator choosing, data availability, and localization. In addition, Ziervogel et al. (2017) addressed the concept of urban resilience, including both the New Urban Agenda and 100 Resilient City Program, and claim that refocusing resilience is needed in order to deal with siloed approach, inequities, and path-dependence, thus taking emphasis on negotiated resilience, endogenous approximation and entitlement is essential. Three articles focused on green infrastructure; nevertheless, only one paper (Meerow and Newell, 2017) proposed a framework for urban planning which eases the identification of high priority areas for implementing green infrastructure and addresses trade-offs and synergies. Lastly, one article focuses on smart city assessment however takes only marginal emphasis on resilience.

2018

In 2018 similarly to the previous years, green infrastructure, ecosystem services, the concept of resilience and sustainability, and urban planning were in the centrum of the most cited studies. Zhang and Li (2018) reviewed the literature and highlighted differences between urban resilience and urban sustainability regarding research trends, focus, and clusters. They presented a framework in which resilience is considered passive while sustainability is active. They concluded that these paradigms should be regarded jointly to plan rationally. Among green infrastructure studies, two used quantitative methodology to connect urban heat island changes to different types of green infrastructures or landcover changes; three articles focused on ecosystem services. The last three studies consider spatial/urban planning perspectives in connection with rural restructuring in the face of rapid urbanization, the management challenges arising from consideration of forest landscapes as social-ecological systems, and in connection with excessive information, communication, and technological focus of the smart city concept.

2019

In 2019 only one top-cited article analyzed the relationship between characteristics of urban green infrastructure (e.g., size, form, distribution) and UHI magnitude, which compared different green space planning traditions. The main emphasis was predominantly on practice; thus, evaluation of applied adaptation/ sustainable solutions, their (conceptual) consequences on urban resilience, and impedimental factors of implementations are prevailed. Meerow and Newell (2019) concentrated on social and equity aspects of resilience planning presented a framework to identify trade-offs inherited political and scalar difficulties. Similarly, Bush and

Doyon (2019) developed a method to help urban planners address (temporal, scale, functional, social equity, and species) trade-offs, but they focused on nature-based solutions. Piggott-McKellar et al. (2019) explored the barriers - rooted in socio-political, resource, and physical systems/processes – which occurred during the implementation of community-based adaptation projects in the Global South that the barriers are similar to those in the academic literature. These three studies included examining social equity, one of the most significant critics regarding urban resilience; however, they applied a more practice-oriented approach compared to the previous years. Nevertheless, Long & Rice (Long & Rice, 2019) dealt with the theoretical concept of climate urbanism (as the original root of urban resilience) and criticized its political context, insufficient social attention, and infrastructure focus since it diverges from sustainability. Finally, von Wirth et al. (2019) explored the mechanisms of the spread of urban living labs' impacts and their contribution to sustainability; and Sharifi (2019) examined which urban structures contribute to resilience.

2020

The most cited papers of 2020 represent a strong body of green infrastructure research. We found quantitative studies measuring the cooling effects of blue-green infrastructures and tree shading, while other studies analyzed the role of green infrastructure in urban planning. On the other hand, Loughran (2020) highlights from a historical perspective how city planners have used green areas to address not only the economic but socio-cultural difficulties of cities. Ugolini et al. (2020) explored the critical drivers of normal green space usage. They discovered that social isolation during the Covid-19 pandemic resulted in behavior changes in green infrastructure visitation and its motivations. As a consequence of the recent substantial attention on green infrastructure, two review articles appeared among the most cited ones, Yu et al. (2020) summarized the literature on blue-green spaces’ cooling effect, while Chatzimentor et al. (2020) revealed the research patterns in the EU and found a high focus on metropolitan regions, a considerable interest in ecosystem services, and insufficient attention on social factors. One of the last two articles is concerned about the global networks and interconnectedness of the adaptation interventions (Goh, 2020), while the other examines urban morphology and street networks but respects resilience scantly (Boeing, 2020).

2021

In 2021, Covid-19 induced studies were outstanding, and economic resilience also came into the scene. McCartney et al. (2021) examined economic and tourism recovery after the lock-down in Macao and identified the economic environment as a crucial precursor of revival; even though they regarded resilience as equivalent to bouncing back, they noted that the short-term recovery urgency and profit-seeking fail to address previous vulnerabilities. In the face of the epidemic, a radical shift is needed in urban planning both from the aspect of urban agriculture, which has lost its significance regardless of its potential to contribute to resilience and sustainability as a nature-based multifunctional solution (Langemeyer et al., 2021) and from the angle of proximity and accessibility to address the concept of 15-Minute City (Moreno et al., 2021). Following the resilience and urban planning line, Olazabal and Gopegui (2021)

evaluated port cities' adaptation strategies for economic and technological credibility and legitimacy. They stated that existing planning requires substantial improvement to be effective, implemented, and achieve long-term sustainability.

LOCK-IN

2015

In the first analyzed year, the selected publications focused mainly on path dependency or lock-in areas: housing policy, infrastructure, and regional development case studies. Housing system and policy were in the focus of Stephens et al. (2015) from a considerable infrastructure point of view since a built-up environment can contribute to the existence of path-dependent decisions. Besides, Filion (2015) argued the strong connection between housing policy regarding suburban areas and potential path-dependent future terms. The second group of the top-cited papers moved around several infrastructure-related issues: considering and integrating long-term impacts of infrastructure development initiatives in terms of potential path-dependency were discussed by Malekpour et al. (2015). A regional point-of-view was applied by many who argued the competitiveness of a given cluster or region regarding path-dependent past and future.

2016

The most cited papers regarding path-dependency and lock-in issues from 2016 indicated more heterogeneity of the analyzed topics. Besides housing studies, the second relevant group of selected articles focused on transitions and transformations: Wolfram (2016) defined urban transformative capacity, including ten components, since transformative change require awareness regarding path dependences and cross-scale linkages; Gouldson et al. (2016) analyzed low emission development strategies by focusing on potential lock-ins and coordination between institutions; Affolderbach and Schulz (2016) encouraged to integrate urban geography into transition studies in order to identify and analyze various forms of synergies; finally, Sorensen (2016) studied periurbanization processes through the lens of path dependencies and stated that newly built-up areas need not only infrastructural background but also institutional agents as well which can contribute to the occurrence of long-term lock-ins. The third group was formulated around regional aspects. Finally, the first climate-related study which dealt with potential lock-ins was written by Bouzarovski et al. (2016), who focused on energy poverty issues as a potentially path-dependent sector. According to their assumptions, various socio-technical lock-ins can be defined through the energetically ineffective residential buildings and the outdated energy supply system. The interlinked mitigation issues make this study one of the first top-cited articles focused on thematic locked-in trajectories.

2017

In 2017, the previously introduced topics were in the focus of top-cited articles again (housing policy, regional clusters, and development), with some relevant exceptions. The most climate-related lock-in analysis was developed by Bouzarovski and Tirado Herrero (2017) regarding

energy poverty. According to Kaika (2017), finding path dependencies have a pivotal role instead of focusing on different adjectives regarding the planned cities in the future, such as safe, sustainable, resilient, and inclusive. This latter study dealt with climate-related aspects in a more detailed way.

2018

Housing studies applied path-dependency analysis in the case of a considerable number of top-cited papers in 2018 similarly to the previous years by applying relevant social aspects to illustrate long-term negative tendencies in various urban areas; moreover, regional-level assessments were also involved in the most cited studies this year. Besides, local-specific studies in connection with lock-in tendencies can be found amongst the topics of selected studies as well. Sorensen (2018) warned that institutional lock-ins could be identified and revealed when “frozen” institutions hinder the development process regarding continuously changing and transforming urban areas. Similarly, institutional aspects were in focus in Hein (2018) by emphasizing the role of a given sector on local path dependencies when locked-in institutions and infrastructure elements can burden further urban transformations. Finally, only one selected article dealt with climate-related issues regarding lock-in analysis: Radhakrishnan et al. (2018) outlined the role of flexibility in adaptation planning processes in the case of water-sensitive cities.

2019

In 2019, a substantial portion of studies related to path dependency was concerned with the housing market. Regarding regional economic studies, smart specialization was criticized (Hassink and Gong, 2019), and literature on evolutionary economic geography was scoped (Zhu et al., 2019). Finally, one paper dealt with return migration in China where rural development is characterized by path dependence and agriculture embedded as the primary source of livelihood (Chen and Wang, 2019), and Davidson et al. (2019) studied the concept of social-ecological resilience in two cities’ plans and state there is a limited acquirement.

2020

Studies in 2020 were concentrated around urban governance and urban development patterns. Tightly connected to resilience, 1) an urban planning tool is proposed to reveal different resilience paths based on local aspects to rectify the embedded planning traces (Wardekker et al., 2020), 2) the concept of resilience resistance is introduced referring to the institutional barriers that are created by way of usual operations but hinder the obtainment of resilience through fatigue, complacency and overconfidence (Shamsuddin, 2020), and 3) the impacts of globalism are explored on adaptation actions (namely flood protection) (Goh, 2020). Ohashi and Phelps (2020) tried to detect the path-dependent mechanism in the restructuring of Tokyo’s suburban areas, Jiao et al. (2020) analyzed the spatial-temporal patterns of urban land-use efficiency quantitatively and referred to changelessness as path-dependence.

2021

The most relevant articles from this year addressed housing issues from the institutional aspects examining the live-work mix (Uyttebrouck et al., 2021), ongoing inadequate interventions dealing with rapid population growth and urbanization (Alaedini and Yeganeh, 2021), or the unintended consequences of decision-making tool which relies on business-as-usual methods, therefore, may cause path-dependency (Schindler and Dionisio, 2021). The second research cluster is concerned with evolutionary spatial economic patterns. Distinctly from the categories above, Ramos (2021) connects resilience and lock-in and argues that the path-shaping notions determine the resilience of Savanna's port city. Finally, we emphasize a pivotal advance taken in 2021. Two studies acknowledged that lock-in could be both harmful and positive as well (Alaedini and Yeganeh, 2021; Zhou and Hu, 2021), and one addressed the positive lock-in phenomenon indirectly (Ramos, 2021).

S3. Preferred Reporting Items for Systematic reviews and Meta-Analyses extension for Scoping Reviews (PRISMA-ScR) Checklist

SECTION	ITEM	PRISMA-ScR CHECKLIST ITEM	REPORTED ON PAGE #
**TITLE**			
Title	1	Identify the report as a scoping review.	1
**ABSTRACT**			
Structured summary	2	Provide a structured summary that includes (as applicable): background, objectives, eligibility criteria, sources of evidence, charting methods, results, and conclusions that relate to the review questions and objectives.	1
**INTRODUCTION**			
Rationale	3	Describe the rationale for the review in the context of what is already known. Explain why the review questions/objectives lend themselves to a scoping review approach.	2-3
Objectives	4	Provide an explicit statement of the questions and objectives being addressed with reference to their key elements (e.g., population or participants, concepts, and context) or other relevant key elements used to conceptualize the review questions and/or objectives.	2-3
**METHODS**			
Protocol and registration	5	Indicate whether a review protocol exists; state if and where it can be accessed (e.g., a Web address); and if available, provide registration information, including the registration number.	3
Eligibility criteria	6	Specify characteristics of the sources of evidence used as eligibility criteria (e.g., years considered, language, and publication status), and provide a rationale.	3-4
Information sources	7	Describe all information sources in the search (e.g., databases with dates of coverage and contact with authors to identify additional sources), as well as the date the most recent search was executed.	3-5
Search	8	Present the full electronic search strategy for at least 1 database, including any limits used, such that it could be repeated.	4
Selection of sources of evidence	9	State the process for selecting sources of evidence (i.e., screening and eligibility) included in the scoping review.	4
Data charting process	10	Describe the methods of charting data from the included sources of evidence (e.g., calibrated forms or forms that have been tested by the team before their use, and whether data charting was done independently or in duplicate) and any processes for obtaining and confirming data from investigators.	5
Data items	11	List and define all variables for which data were sought and any assumptions and simplifications made.	5
Critical appraisal of individual sources of evidence	12	If done, provide a rationale for conducting a critical appraisal of included sources of evidence; describe the methods used and how this information was used in any data synthesis (if appropriate).	-
Synthesis of results	13	Describe the methods of handling and summarizing the data that were charted.	5
**RESULTS**			

SECTION	ITEM	PRISMA-ScR CHECKLIST ITEM	REPORTED ON PAGE #
Selection of sources of evidence	14	Give numbers of sources of evidence screened, assessed for eligibility, and included in the review, with reasons for exclusions at each stage, ideally using a flow diagram.	6
Characteristics of sources of evidence	15	For each source of evidence, present characteristics for which data were charted and provide the citations.	5-13 and S1.
Critical appraisal within sources of evidence	16	If done, present data on critical appraisal of included sources of evidence (see item 12).	-
Results of individual sources of evidence	17	For each included source of evidence, present the relevant data that were charted that relate to the review questions and objectives.	6-13 and S2.
Synthesis of results	18	Summarize and/or present the charting results as they relate to the review questions and objectives.	6-13
**DISCUSSION**			
Summary of evidence	19	Summarize the main results (including an overview of concepts, themes, and types of evidence available), link to the review questions and objectives, and consider the relevance to key groups.	13-14
Limitations	20	Discuss the limitations of the scoping review process.	14
Conclusions	21	Provide a general interpretation of the results with respect to the review questions and objectives, as well as potential implications and/or next steps.	14
**FUNDING**			
Funding	22	Describe sources of funding for the included sources of evidence, as well as sources of funding for the scoping review. Describe the role of the funders of the scoping review.	1

From: Tricco AC, Lillie E, Zarin W, O'Brien KK, Colquhoun H, Levac D, et al. PRISMA Extension for Scoping Reviews (PRISMAScR): Checklist and Explanation. Ann Intern Med. 2018;169:467–473. doi: 10.7326/M18-0850.
